# Clinical impact of obsessive-compulsive disorder comorbidity in bipolar disorder: a systematic review and meta-analysis: OCD in BD: A Review on Clinical Impact

**DOI:** 10.1192/j.eurpsy.2025.10087

**Published:** 2025-08-26

**Authors:** Michele De Prisco, Cristiana Tapoi, Vincenzo Oliva, Robertas Strumila, Christine Takami, Nicolaja Girone, Monica Macellaro, Juliana Braga de Salles Andrade, Christian Nikolaus Schmitz, Eduard Vieta, Giovanna Fico

**Affiliations:** 1 Institute of Neurosciences (ICN), Department of Medicine, Faculty of Medicine and Health Sciences, Universitat de Barcelona (UB), Barcelona, Spain; 2 Bipolar and Depressive Disorders Unit, Hospìtal Clinic de Barcelona, Barcelona, Spain; 3 Bipolar and Depressive Disorders Unit, Institut d’Investigacions Biomèdiques August Pi i Sunyer (IDIBAPS), Barcelona, Spain; 4 Centro de Investigación Biomédica en Red de Salud Mental (CIBERSAM), Instituto de Salud Carlos III, Madrid, Spain; 5 Department of General Psychiatry, Prof. Dr. Alexandru Obregia Clinical Psychiatry Hospital, Bucharest, Romania; 6 Department of Urgent and Post Urgent Psychiatry, CHU Montpellier, Montpellier, France; 7 Institute of Functional Genomics, University of Montpellier, CNRS, INSERM, Montpellier, France; 8 Faculty of Medicine, Institute of Clinical Medicine, Psychiatric Clinic, Vilnius University, Vilnius, Lithuania; 9 Department of Medical Epidemiology and Biostatistics, Karolinska Institutet, Stockholm, Sweden; 10 Department of Biomedical and Clinical Sciences “Luigi Sacco”, Department of Psychiatry, University of Milan, Milan, Italy; 11 Brain Connectivity Unit, D’Or Institute for Research and Education - IDOR, Rio de Janeiro, Brazil; 12 Department of Molecular Neuroimaging, Central Institute of Mental Health, Medical Faculty Mannheim, Heidelberg University, Mannheim, Germany; 13 Department of Psychiatry and Psychotherapy, Central Institute of Mental Health, Medical Faculty Mannheim, Heidelberg University, Mannheim, Germany; 14 German Center for Mental Health (DZPG), Partner Site Mannheim, Mannheim, Germany

**Keywords:** bipolar disorder, comorbidity, meta-analysis, obsessive-compulsive disorder, suicide

## Abstract

**Background:**

Bipolar disorder (BD) is commonly comorbid with other psychiatric conditions, such as obsessive-compulsive disorder (OCD). Despite increasing interest in this comorbidity, quantitative data on its clinical characteristics remain limited. This systematic review and meta-analysis aimed to evaluate the clinical impact of OCD comorbidity in BD by comparing individuals with BD and OCD (BD-OCD) to those with BD without OCD.

**Methods:**

We systematically searched the PubMed/MEDLINE, Scopus, PsycINFO, and Web of Science databases up to April 15, 2024. Meta-analyses were conducted to compare BD-OCD and BD without OCD groups across multiple clinical domains.

**Results:**

From 11,959 initial records screened, 26 studies were included in the qualitative synthesis, with 22 eligible for meta-analysis. Individuals with BD-OCD showed higher odds of experiencing chronic mood episodes (OR = 9.42; 95%CI = 2.23, 39.9), rapid cycling (OR = 1.92; 95%CI = 1.04, 3.53), comorbid eating disorders (OR = 3.37; 95%CI = 1.99, 5.7), panic disorder (OR = 3.3; 95%CI = 2.11, 5.2), substance use disorders (OR = 1.39; 95%CI = 1.02, 1.89), and lifetime suicide attempts (OR = 1.85; 95%CI = 1.21, 2.84). Additionally, they presented earlier onset of BD (SMD = -0.27; 95%CI = -0.52, −0.01) and reduced functioning (SMD = -0.42; 95%CI = -0.59, −0.24). Most data were derived from adult populations, limiting the evidence available for children and adolescents.

**Conclusions:**

BD-OCD presents a more severe and complex clinical profile, requiring specialized assessment and integrated treatment approaches. Identifying these features may support earlier recognition and inform personalized interventions for this population.

## Introduction

Bipolar disorder (BD) is a chronic mood disorder with a lifetime prevalence between 0.4 and 1.1%, characterized by a clinical course of recurrent depressive, hypomanic, or manic episodes alternating with periods of euthymia [1]. Beyond its symptomatic burden, BD significantly impacts quality of life through persistent functional and cognitive impairments [2–4]. Life expectancy is often shortened due to increased suicide risk [5] and common co-occurring conditions such as cardiovascular disease [6] and metabolic syndrome [7]. The clinical picture becomes even more complex when BD co-occurs with other psychiatric conditions. Indeed, these patients commonly experience comorbid anxiety disorders, obsessive-compulsive disorder (OCD), substance use disorders, attention-deficit/hyperactivity disorder, eating disorders, or personality disorders, creating complex clinical presentations that require comprehensive treatment approaches [8].

The comorbidity between BD and OCD requires special consideration, as population-based studies reveal lifetime rates of OCD in BD ranging from 0.26 to 27.8% [9]. Familial aggregation data show that first-degree relatives of probands with OCD have an increased risk of BD, with this risk diminishing as genetic distance increases [10], suggesting a shared genetic diathesis. Clinically, OCD in BD frequently follows an episodic, mood-dependent pattern, with subjects experiencing OCD symptoms mostly during depressive episodes or reporting symptom worsening during these periods [9]. This comorbidity also presents important treatment challenges. Antidepressants, which are commonly used as first-line treatments for OCD [11], should be used with caution in BD due to the risk of triggering manic episodes [12]. Recognizing this comorbidity is crucial for optimizing patient management and ensuring a balanced therapeutic approach. However, antidepressants can be safely used as add-on therapy in BD, and their use is supported by clinical guidelines [13] when properly monitored and combined with mood stabilizers [14, 15], potentially offering relief to individuals struggling with both conditions. Beyond presenting a therapeutic challenge, the comorbidity of BD and OCD complicates clinical management due to its complex presentation, which may involve a higher number of comorbidities and a more severe clinical course [9, 16, 17].

In a previous study [18], we examined phenomenological and clinical differences in obsessive-compulsive symptomatology between individuals with BD and OCD and those with OCD alone. We found that patients with the comorbid condition were more likely to experience sexual obsessions and less likely to report contamination obsessions, with no significant differences in overall symptom severity. While that work contributed to a better understanding of the obsessive-compulsive symptom profile of comorbid BD and OCD, it did not address a central clinical question on how OCD influences the course of BD itself.

The present study addresses this gap by shifting the focus to individuals with BD, comparing those with comorbid OCD (BD-OCD) to those without.

Our goal is to provide a detailed clinical characterization of this comorbidity by examining various features, including symptom severity, characteristics of affective episodes, history of suicide attempts, presence of additional psychiatric disorders, and overall functional status. Understanding these differences is essential for refining treatment strategies and improving the quality of life of people experiencing these disorders.

## Material and methods

This systematic review and meta-analysis were conducted according to the Preferred Reporting Items for Systematic Reviews and Meta-Analyses (PRISMA) guidelines [19]. The protocol was registered on the International Prospective Register of Systematic Reviews (PROSPERO) (https://www.crd.york.ac.uk/PROSPERO/; protocol CRD42024536387), and any deviation is reported in the Supplementary Materials, Appendix I.

### Search strategy

We searched the PubMed/MEDLINE, Scopus, PsycINFO, and Web of Science databases from inception to April 15th, 2024. The search strategies are detailed in the Supplementary Materials, Appendix II. To identify potential additional studies not captured by the original search string, the references of each included study, textbooks, and other materials were additionally searched.

### Eligibility criteria and study outcomes

We included original studies providing quantitative data about the differences between people diagnosed with BD-OCD, and people diagnosed with BD alone on specific clinical outcomes: (a) the scores obtained at validated scales measuring clinical symptoms severity (e.g., affective symptomatology, anxiety symptomatology, functioning), (b) number and duration of lifetime affective episodes, (c) number of previous hospitalization for psychiatric conditions, (d) age at onset for the BD, (e) duration of BD, (f) presence of other psychiatric comorbidities, (g) suicidality, and (h) characteristics of affective episodes (e.g., psychotic features, melancholic features, rapid cycling). Psychiatric diagnoses were required to be made according to the Diagnostic and Statistical Manual for Mental Disorders (DSM) or the International Classification of Diseases (ICD) [20] diagnostic criteria. There were no restrictions on language or age. Both observational and interventional studies were considered for inclusion: for longitudinal observational studies, baseline data were prioritized; if unavailable, data from the first follow-up were used. For interventional studies, only baseline data were included. In instances where multiple studies had overlapping populations, the largest study with the most representative data relevant to our objectives was selected. We excluded reviews, case reports, and case series, and studies conducted on animals or on human populations with diagnoses outside the scope of our criteria.

### Study selection and data extraction

Four groups of two authors each (MDP and CNS, CT and JBdSA, RS and CTL, NG and MM) independently reviewed studies of potential interest, and a third author (VO or GF) was consulted when a consensus could not be reached. Data extraction was independently conducted by two authors (MDP and CT) and included (when available): first author, publication year, study design, geographical region, country, diagnostic criteria considered, (semi)structured interview adopted, number of cases (i.e., people with BD-OCD) included in the study, number of controls (i.e., people with BD alone) included in the study, mean and standard deviation (SD) for the outcomes measured continuously (e.g., clinical symptoms severity, age at onset, duration of illness), and number of events and non-events for the outcomes measured dichotomously (e.g., presence of other psychiatric comorbidities, characteristics of affective episodes), mean age, % of females, BD type, affective state (i.e., % of people in euthymia, depression, (hypo)mania, or mixed state), medical comorbidities, and treatment for cases and controls (current or lifetime). When information was not available, we contacted the authors to request the relevant data.

### Risk of bias

Two authors (NG and MM) independently evaluated the risk of bias in the included studies, with a third author (MDP or VO) resolving any disagreements. The Newcastle-Ottawa Scale (NOS) [21] was used to rate the quality of observational studies, and NOS scores were converted to “Agency for Healthcare Research and Quality” (AHRQ) standards, as done elsewhere [22].

### Statistical snalyses

We conducted random-effect meta-analyses (restricted maximum-likelihood estimator) [23] through the R-package “metafor” [24], using R, version 4.3.1 [25]. For continuous outcomes, standardized mean differences (SMD), represented by Hedge’s g with corresponding confidence intervals (CIs), were used as effect size. For dichotomous outcomes, odds ratios (OR) with corresponding CIs were used as the effect size. We graphically presented our results using a jungle plot [26], where the x-axis represents the effect sizes (expressed as SMD or logOR) and the y-axis displays the various comparisons analyzed. Sensitivity analyses were performed by (a) excluding one study at a time from the main analysis (leave-one-out), and (b) including only high-quality studies as defined by AHRQ standards. We assessed heterogeneity by using Cochran’s *Q* test [27], and *I*^2^ statistics [28]. When the Cochran’s *Q* test presented a *p* < 0.10 or the *I*^2^ statistic showed a value >50%, and when at least ten studies providing this information were available, meta-regression analyses were conducted. Predictors included: mean age, percentage of females, percentage of individuals in euthymia, depression, (hypo)mania, or mixed symptoms, percentage with BD-I, and percentage taking specific medication classes. Prediction intervals were calculated. Publication bias was explored by visually examining funnel plots and using Egger’s test [29] when at least ten studies were included in the analysis.

## Results

**Figure 1.**
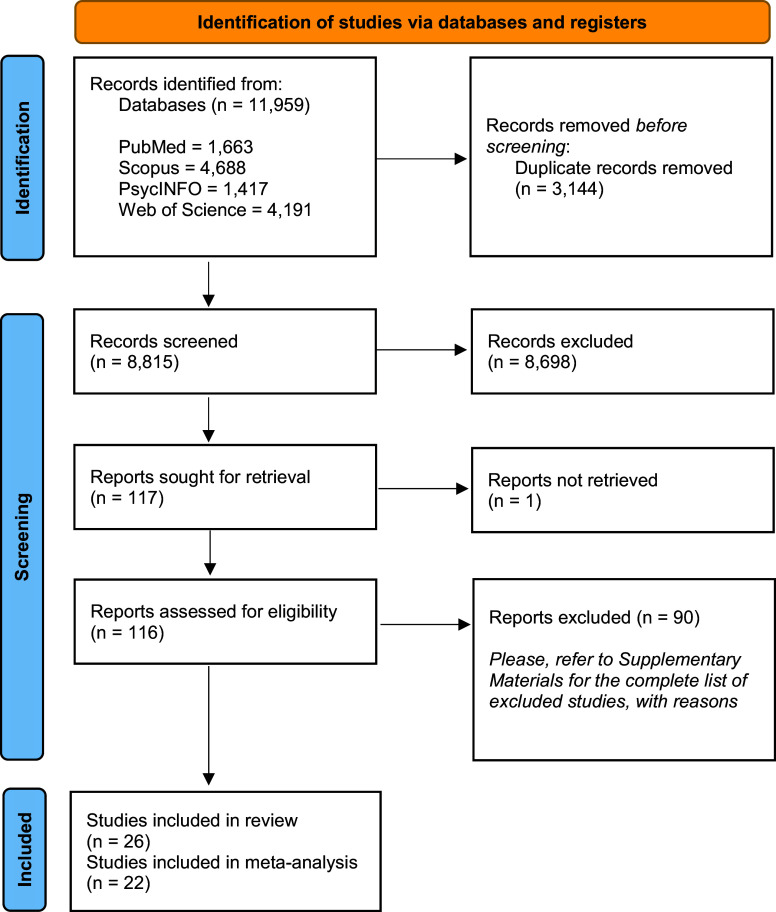
PRISMA flowchart, 2020 edition, adapted.

A total of 11,959 references were identified from various sources. After duplicate removal, 8,698 studies were excluded at the title/abstract level, 90 after the full-text evaluation, and one study was not retrieved. Finally, we included 26 studies in this systematic review [16, 17, 30–53], of which 22 [16, 17, 30–34, 37–42, 44–49, 51–53] provided sufficient data to perform a meta-analysis. The PRISMA flowchart is reported in Figure 1. The studies excluded from this review are detailed in the Supplementary Materials, Appendix III.

### Characteristics of included studies

The 26 studies included were published between 1995 and 2023. The total number of people with BD-OCD was 1,095 (range = 6–201) compared to 5,583 (range = 15–1,613) people with BD. Twenty-two studies were cross-sectional, and four were prospective-cohort studies. Twenty studies focused on adult patients, four included children/adolescents, and two considered both adults and children/adolescents in their samples.

Individuals diagnosed with BD-OCD (54.8% female) had a mean age of 34.65 years (±9.6). Fourteen studies reported information about the type of BD; among these, 55.6% (range = 0–100) of the included patients were diagnosed with BD type I. Regarding mood state, eight studies provided data: 54.2% of the sample were euthymic, 21% were depressed, 20% were (hypo)manic, and 4.8% experienced a mixed episode.

Onset sequence was reported in 12 studies (46%). Of these, seven studies (27%) provided detailed information about the percentage of individuals in whom BD preceded OCD, OCD preceded BD, or both disorders emerged simultaneously. The remaining five studies (19%) reported only the mean age at onset for each disorder, without additional details regarding onset sequence.

Individuals diagnosed with BD without OCD (57.8% female) had a mean age of 40.31 years (±7.84). Fourteen studies reported information about the type of BD; among these, 71.8% (range = 0–100) of the included patients were diagnosed with BD type I. Regarding mood state, eight studies provided data: 65.1% of the sample were euthymic, 24.2% were depressed, 7.5% were (hypo)manic, and 3.2% experienced a mixed episode.

Additional information on the studies included in the systematic review and meta-analysis is presented in Table 1, while additional information on the onset sequence is available in the Supplementary Materials, Appendix IV.

**Table 1.** Characteristics of the studies included in the systematic review and meta-analysis

References, country	Study design	Population (*n*)	Mean age	Percentage of females	Diagnostic criteria	Outcome type	NOS (AHRQ)
Bener et al. [30], Qatar	Cross-sectional	BD-OCD (92) BD (304)	43.3 ± 13.3 41.4 ± 12.3	58.4% 59.5%	DSM-IV	Clinical symptom severity; hospitalization, number; age at onset of BD; duration of BD; presence of other psychiatric comorbidities	7 (GOOD)
Braveman et al. [31], Israel	Cross-sectional	BD-OCD (19) BD (37)	33.6 ± 10.4 33.1 ± 10.4	68.4% 48.6%	DSM-5	Clinical symptom severity; age at onset of BD	5 (POOR)
Centorrino et al. [32], USA	Longitudinal	BD-OCD (16) BD (21)	33.7 ± 10.3 35.3 ± 9.4	36% 59%	DSM-IV	Clinical symptom severity; suicidality	7 (FAIR)
Chen et al. [33], USA	Cross-sectional	BD-OCD (35) BD (132)	NA NA	NA NA	DSM-III	Age at onset of BD; presence of other psychiatric comorbidities; suicidality	4 (POOR)
De Filippis et al. [34], Italy	Cross-sectional	BD-OCD (26) BD (22)	47.4 ± 13.2 47.9 ± 11.8	69% 50%	DSM-5	Clinical symptom severity; duration of BD; characteristics of affective episodes	7 (GOOD)
Di Salvo et al. [16], Italy	Cross-sectional	BD-OCD (201) BD (789)	42.3 ± 14.1 50.7 ± 15.5	48% 60%	DSM-IV-TR DSM-5	Age at onset of BD; presence of other psychiatric comorbidities; suicidality	4 (POOR)
Dilsaver et al. [35], USA	Cross-sectional	BD-OCD (54) BD (61)	NA NA	NA NA	DSM-IV	Suicidality	5 (POOR)
Ezzat et al. [36], Egypt	Cross-sectional	BD-OCD (26) BD (54)	19.9 ± 1.9 22 ± 1.1	54% 41%	DSM-5	Clinical symptom severity; age at onset of BD; suicidality	5 (POOR)
Goes et al. [17], USA	Cross-sectional	BD-OCD (89) BD (1613)	36.6 ± 1.1 42.1 ± 0.7	75% 63%	DSM-III-R DSM-IV	Presence of other psychiatric comorbidities; suicidality; characteristics of affective episodes	5 (POOR)
Goodwin et al. [37], USA	Cross-sectional	BD-OCD (6) BD (27)	NA NA	NA NA	DSM-III-R	Presence of other psychiatric comorbidities	5 (POOR)
Hassani et al. [38], Iran	Cross-sectional	BD-OCD (25) BD (25)	28.9 ± 5.4 29.1 ± 5.2	48% 44%	DSM-IV	Clinical symptom severity; suicidality	6 (GOOD)
Issler et al. [39], Brazil	Cross-sectional	BD-OCD (15) BD (15)	38.9 ± 10.7 41.8 ± 10.5	100% 100%	DSM-IV	Hospitalization, number; age at onset of BD; duration of BD; presence of other psychiatric comorbidities; suicidality; characteristics of affective episodes	7 (GOOD)
Jeon et al. [40], Korea	Cross-sectional	BD-OCD (50) BD (284)	31.8 ± 9.7 35.5 ± 11.9	62% 66%	DSM-IV	Age at onset of BD; duration of BD; presence of other psychiatric comorbidities; suicidality; characteristics of affective episodes	5 (POOR)
Joshi et al. [41], USA	Cross-sectional	BD-OCD (17) BD (65)	10.5 ± 2.9 10.0 ± 3.8	18% 26%	DSM-III-R	Clinical symptom severity; age at onset of BD; duration of BD; presence of other psychiatric comorbidities	5 (POOR)
Kazhungil et al. [42], India	Cross-sectional	BD-OCD (32) BD (58)	41 ± 11.2 39.4 ± 11.2	75% 64%	DSM-IV	Clinical symptom severity; hospitalization, number; age at onset of BD; suicidality; characteristics of affective episodes	8 (GOOD)
Khan et al. [43], Pakistan	Cross-sectional	BD-OCD (35) BD (434)	NA NA	50% 50%	DSM-IV-TR	Duration of BD	7 (GOOD)
Kim et al. [44], Australia	Longitudinal	BD-OCD (24) BD (150)	39.8 ± 12 43 ± 13.6	71% 59%	DSM-IV	Clinical symptom severity; age at onset of BD; suicidality	7 (GOOD)
Kocabas et al. [45], Turkey	Cross-sectional	BD-OCD (28) BD (172)	NA NA	NA NA	DSM-IV	Suicidality	8 (GOOD)
Koyoncu et al. [46], Turkey	Cross-sectional	BD-OCD (35) BD (35)	36.2 ± 15.9 34.8 ± 10.3	60% 69%	DSM-IV	Age at onset of BD; presence of other psychiatric comorbidities; suicidality; characteristics of affective episodes	8 (GOOD)
Kruger et al. [47], Germany	Cross-sectional	BD-OCD (10) BD (133)	44.1 ± 12.7 44.03 ± 14	0% 68%	DSM-III	Age at onset of BD; presence of other psychiatric comorbidities; suicidality; characteristics of affective episodes	6 (POOR)
Levander et al. [48], multicenter (Germany, The Netherlands, USA)	Cross-sectional	BD-OCD (48) BD (302)	NA NA	NA NA	DSM-IV	Presence of other psychiatric comorbidities	5 (POOR)
Magalhaes et al. [49], Brazil	Cross-sectional	BD-OCD (32) BD (227)	41.4 ± 12.8 41.7 ± 11.7	84% 68%	DSM-IV	Duration of BD; presence of other psychiatric comorbidities; suicidality; characteristics of affective episodes	5 (POOR)
Masi et al. [50], Italy	Longitudinal	BD-OCD (30) BD (37)	14.2 ± 3.1 14.6 ± 3.4	40% 43%	DSM-IV	Clinical symptom severity; age at onset of BD; presence of other psychiatric comorbidities	8 (GOOD)
Masi et al. [53], Italy	Longitudinal	BD-OCD (88) BD (172)	14.2 ± 2.6 13.7 ± 2.9	36% 40%	DSM-IV	Clinical symptom severity; age at onset of BD; presence of other psychiatric comorbidities; characteristics of affective episodes	5 (POOR)
Ozdemiroglu et al. [51], Turkey	Cross-sectional	BD-OCD (32) BD (48)	36.2 ± 12 42.3 ± 12.4	50% 52%	DSM-IV	Clinical symptom severity, age at onset of BD; and characteristics of affective episodes	7 (GOOD)
Shashidhara et al. [52], India	Cross-sectional	BD-OCD (30) BD (366)	30.6 ± 11.9 31.8 ± 11.1	37% 42%	DSM-IV-TR	Clinical symptom severity: hospitalization, number; age at onset of BD; duration of BD; presence of other psychiatric comorbidities, characteristics of affective episodes	8 (GOOD)

Abbreviations**:** AHRQ, Agency for Healthcare Research and Quality; BD, Bipolar Disorder; DSM, Diagnostic and Statistical Manual of Mental Disorders; NOS, Newcastle-Ottawa Scale; OCD, Obsessive-Compulsive Disorder.

### Risk of bias evaluation

Twelve studies were rated as “good quality” according to the AHRQ standards, one study was rated as “fair quality,” and 13 studies were rated as “poor quality.” Details are provided in Table 1 and in the Supplementary Materials, Appendix IV.

### Main analyses

**Figure 2.**
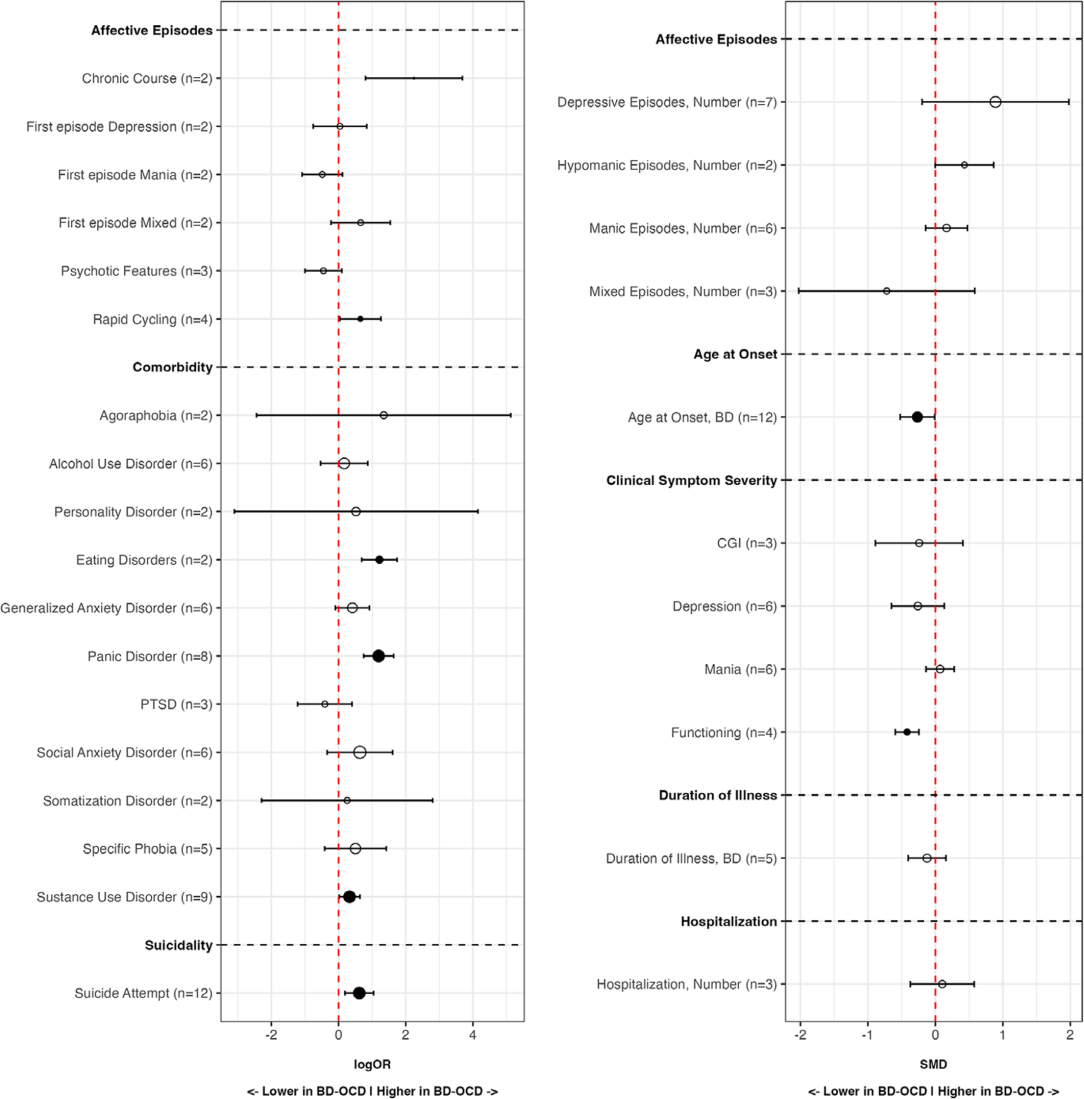
Jungle plot for differences between adults with BD-OCD and adults with BD. Black-filled circles indicate statistically significant comparisons, while white-filled ones indicate non-significant comparisons. Point size is proportional to the number of patients included in that specific comparison. logOR has been used instead of OR for graphical purposes. logOR >0 reflects higher odds in the BD-OCD group. Legend: BD, Bipolar Disorder; CGI, Clinical Global Impression; OCD, Obsessive-Compulsive Disorder; PTSD, Post-Traumatic Stress Disorder.

The main results of the meta-analyses conducted are displayed in Table 2 and Figure 2.

**Table 2.** Results of the meta-analyses in detail

Outcome type	Studies, n	BD-OCD patients, *n*	BD patients, n	Effect size, type	Effect size	95% CIs	*p*-value	95% PIs	***I***^**2**^	Q test *p*-value
*Adults*										
*Affective episodes*										
Chronic course	2	50	50	OR	**9.422**	**2.228, 39.885**	**0.002**	**2.228, 39.885**	0	0.7
First episode depression	2	65	401	OR	1.042	0.47, 2.314	0.92	0.47, 2.314	0	0.46
First episode mania	2	65	401	OR	0.616	0.338, 1.124	0.11	0.338, 1.124	0	0.76
First episode mixed	2	65	401	OR	1.931	0.799, 4.665	0.14	0.732, 5.094	10.45	0.29
Psychotic features	3	80	416	OR	0.638	0.368, 1.103	0.11	0.368, 1.103	0	0.61
rapid cycling	4	109	443	OR	**1.916**	**1.04, 3.532**	**0.037**	**1.04, 3.532**	0	0.37
*Comorbidity*										
Agoraphobia	2	122	670	OR	3.838	0.087, 168.511	0.49	0.008, 1835.367	80.89	<0.1
Alcohol use disorder	6	245	2336	OR	1.183	0.586, 2.387	0.64	0.257, 5.458	74.24	<0.1
Any personality disorder	2	293	1093	OR	1.68	0.045, 63.371	0.78	0.003, 860.058	97.64	<0.1
Any eating disorder	2	251	1073	OR	**3.374**	**1.992, 5.709**	**<0.001**	**1.992, 5.709**	0	0.86
Generalized anxiety disorder	6	383	1642	OR	1.51	0.909, 2.507	0.11	0.909, 2.507	0	0.89
Panic disorder	8	522	3539	OR	**3.297**	**2.111, 5.155**	**<0.001**	**1.422, 7.652**	41.52	0.19
PTSD	3	142	354	OR	0.665	0.296, 1.493	0.32	0.296, 1.493	0	0.98
Social anxiety disorder	6	462	3122	OR	1.887	0.712, 5.003	0.2	0.18, 19.787	84.53	<0.1
Somatization disorder	2	102	437	OR	1.292	0.101, 16.461	0.84	0.02, 85.03	84.68	<0.1
Specific phobia	5	241	2100	OR	1.654	0.662, 4.137	0.28	0.272, 10.054	62.08	<0.1
Substance use disorder	9	488	3606	OR	**1.387**	**1.018, 1.891**	**0.038**	0.89, 2.164	11.24	0.31
*Suicidality*										
Suicide, attempt	12	568	3494	OR	**1.853**	**1.21, 2.841**	**0.005**	0.554, 6.209	69.58	<0.1
*Affective episodes*										
Depressive episodes, number	7	274	2406	SMD	0.891	−0.197, 1.979	0.11	−2.149, 3.931	98.12	<0.1
Hypomanic episodes, number	2	62	414	SMD	0.431	−0.003, 0.864	0.05	−0.195, 1.057	53.88	0.14
Manic episodes, number	6	185	793	SMD	0.166	−0.144, 0.475	0.29	−0.506, 0.837	64.95	<0.1
Mixed episodes, number	3	94	472	SMD	−0.721	−2.026, 0.584	0.28	−3.296, 1.853	96.45	<0.1
*Age at onset*										
BD	12	575	2351	SMD	**−0.266**	**−0.522, −0.01**	**0.042**	−1.076, 0.544	82.9	<0.1
*Clinical symptom severity*										
CGI	3	138	691	SMD	−0.24	−0.889, 0.408	0.47	−1.462, 0.982	87.95	<0.1
Depression	6	215	808	SMD	−0.26	−0.651, 0.132	0.19	−1.182, 0.662	79.51	<0.1
Mania	6	215	808	SMD	0.07	−0.139, 0.279	0.51	−0.28, 0.419	30.81	0.24
Functioning, GAF	4	170	749	SMD	**−0.419**	**−0.594, −0.244**	**<0.001**	**−0.594, −0.244**	0.01	0.46
*Duration of illness*										
BD	5	213	991	SMD	−0.124	−0.403, 0.156	0.39	−0.671, 0.423	61.54	<0.1
*Hospitalization*										
Number	3	154	728	SMD	0.102	−0.372, 0.575	0.67	−0.778, 0.981	82.5	<0.1
*Children/adolescents*										
*Comorbidity*										
Conduct disorder	2	105	237	OR	0.872	0.499, 1.523	0.63	0.499, 1.523	0	0.83
Generalized anxiety disorder	2	105	237	OR	2.067	0.357, 11.965	0.42	0.114, 37.525	85.5	<0.1
Oppositional defiant disorder	2	105	237	OR	1.183	0.695, 2.014	0.54	0.695, 2.014	0	0.59
Panic disorder	2	105	237	OR	1.019	0.452, 2.296	0.96	0.359, 2.892	24.04	0.25
Separation anxiety disorder	2	105	237	OR	1.531	0.907, 2.586	0.11	0.907, 2.586	0	0.55
Social anxiety disorder	2	105	237	OR	1.623	0.974, 2.699	0.06	0.974, 2.699	0	0.8
Tic disorder	2	105	237	OR	3.323	1, 11.034	0.05	0.527, 20.947	66.75	<0.1
*Age at onset*										
BD	2	105	237	SMD	0.111	−0.161, 0.382	0.42	−0.211, 0.432	14.42	0.28

Abbreviations: BD, Bipolar Disorder; CGI, Clinical Global Impression; CI, Confidence Interval; PI, Prediction Interval; OCD, Obsessive-Compulsive Disorder; OR, Odds Ratio; SMD, Standardized Mean Difference. Statistically significant results (p < 0.05) are highlighted in bold.

Overall, adults with BD-OCD showed higher odds of having a mood episode with a chronic course, a rapid cycling course, comorbid eating, panic, and substance use disorder, and a history of suicide attempts when compared to those without OCD. Additionally, adults with BD-OCD presented a younger age at onset of BD and a reduced functioning when compared to people with BD without OCD.

Additional details on the main analyses are presented in the Supplementary Materials, Appendix V.

### Meta-regression analyses

Sociodemographic and clinical characteristics of individuals with BD-OCD were selected as predictors for the meta-regression analyses when there was high heterogeneity and at least ten studies with this information were available.

Specifically, we explored the mean age and percentage of females among individuals with BD-OCD in the comparisons related to the age at onset of BD and lifetime suicide attempts in adults. However, no significant predictors were found.

Additional details on the meta-regression analyses are presented in the Supplementary Materials, Appendix V.

### Sensitivity Analyses

Sensitivity analyses were conducted: (a) by excluding one study at a time from the main analysis, and (b) by including only high-quality studies according to AHRQ standards.

Additional details on the sensitivity analyses are presented in the Supplementary Materials, Appendix V.

### Publication bias

Publication bias was examined in two comparisons with at least ten available studies. Among these comparisons, a significant publication bias was detected for the comparison relative to the age at onset of BD in aduts (Egger Test: z = −1.964; *p*-value = 0.049).

Additional details on the publication bias are presented in the Supplementary Materials, Appendix V.

### Characteristics of the studies and comparisons included in the qualitative synthesis

Four studies [35, 36, 43, 50] were included in the systematic review but were not included in any meta-analysis.

The sample from one of these studies [50] was partially included in another study [53]. However, since the latter study was more recent and had a larger sample size, we decided to include only this one in our analyses.

The remaining three studies [35, 36, 43] provided measures that were not compatible with pooling with any of the other included studies. The first study [35] found that children and adolescents with BD-OCD had higher odds of suicidal ideation compared to those with BD alone. The second study [36], which included both children/adolescents and adults, found that individuals with BD-OCD had an earlier onset of BD, more severe depressive and manic symptoms, and higher scores on scales measuring suicidal ideation. Finally, the third study [43] found no differences in illness duration between individuals with BD-OCD and those without OCD. However, the data were presented in several categories rather than continuously, making it non-comparable with other studies.

## Discussion

This systematic review and meta-analysis aimed to assess the clinical impact of OCD comorbidity in BD by comparing individuals diagnosed with both BD and OCD to those diagnosed with BD without OCD. Our findings revealed significant clinical differences between the groups. Overall, adults with the comorbid condition were more likely to have experienced a mood episode with a chronic or rapid cycling course, have a lifetime comorbidity with eating, panic, or substance use disorders, and have a history of suicide attempts. Furthermore, these patients had a younger age at BD onset and reduced functioning. No significant differences were found in children and adolescents. These findings suggest that the co-occurrence of OCD in adults with BD may reflect a more complex and severe manifestation of affective dysregulation, rather than simply the coexistence of two independent disorders. This clinical profile appears to complicate the illness trajectory of BD, warranting careful assessment and tailored management.

The association between OCD comorbidity and chronic affective episodes reinforces this interpretation. The two studies supporting this finding [39, 46] defined a chronic episode as one in which the criteria for a major mood episode were continuously met for at least two years, resulting in prolonged suffering and functional impairment. The chronicity observed in our sample may not be explained by BD subtype, as previously suggested [54, 55], since the proportion of BD-I and BD-II patients was comparable across the studies contributing to this comparison. Rather, the presence of OCD may serve as a clinical marker of sustained affective instability, potentially complicating both the course of illness and treatment responsiveness. Indeed, OCD comorbidity may contribute to treatment resistance and persistent symptomatology, with prolonged episodes possibly reflecting the complex pharmacological management required when addressing both conditions simultaneously. However, the limited number of studies informing this comparison restricts our ability to draw definitive conclusions about the specific mechanisms underlying the association between OCD comorbidity and chronicity in BD.

A similar argument applies to the increased likelihood of rapid cycling observed in adults with BD-OCD. Rapid cycling, defined as four or more mood episodes within a 12-month period [56], is associated with greater illness severity and poorer outcomes [57]. Its lifetime prevalence in BD is estimated to be around 35% and is linked to several risk factors, including suicidality, childhood maltreatment, female sex, and poor response to mood stabilizers [58]. Antidepressant use, frequently employed as first-line treatment for OCD, has been associated with cycle acceleration in BD [59, 60], though evidence mostly comes from observational studies. In individuals with both BD and OCD, antidepressants may increase the risk of mood instability, potentially contributing to the higher prevalence of rapid cycling observed in our sample. Instead of antidepressants, other treatment options are often considered as first-line for individuals with BD. Common therapies include mood stabilizers and atypical antipsychotics such as lithium, valproic acid, and aripiprazole [1]. Individuals with comorbid BD-OCD likely receive similar treatments, which may mitigate the risk of antidepressant-induced manic switches or mood instability. However, due to limited data in the included studies, we were unable to statistically evaluate the effects of these treatments through meta-regression. A more comprehensive pharmacological profile of this population is needed to better assess its potential impact on rapid cycling. Beyond pharmacological factors, cognitive-affective mechanisms may also play a role. People with BD are known to engage in heightened rumination in response to both negative and positive affective states [3], which is associated with more severe mood symptoms [61, 62]. Similarly, individuals with OCD tend to ruminate extensively on intrusive thoughts and mood-related content. Experimental evidence shows that rumination in OCD maintains both obsessive-compulsive symptoms and depressed mood in the short and intermediate term [63]. In particular, rumination may impede emotional recovery and prolong negative affect. This shared cognitive style may represent a transdiagnostic mechanism that amplifies mood lability and increases vulnerability to affective recurrence, particularly when both disorders are present, thereby contributing to a more unstable clinical course, which requires careful pharmacological management and targeted psychotherapeutic support.

Our meta-analysis revealed higher rates of specific additional comorbidities in adults with BD-OCD. The co-occurrence of BD and eating disorders has been previously documented and is thought to reflect a shared vulnerability related to emotional dysregulation, potentially part of a broader neurodevelopmental continuum [64]. The presence of OCD, which shares traits with eating disorders traits such as perfectionism, reduced executive functioning, and impaired cognitive flexibility, often leads to rigid and repetitive behaviors [65], may account for the increased prevalence of eating disorders in the BD-OCD group. The elevated presence of panic disorder is consistent with the high anxiety burden reported in both BD [66] and OCD [67]. This triple comorbidity pattern suggests a complex interaction between mood, anxiety, and obsessive-compulsive dimensions, possibly reflecting a more severe underlying neurobiological dysregulation. The higher prevalence of substance use disorders in the BD-OCD group likely reflects both a consequence and a contributor to clinical complexity. Individuals may use substances as a means of coping with heightened distress and impairment associated with the comorbidity [68], while substance use itself may further destabilize mood and exacerbate the course of illness [69]. The co-occurrence of these conditions may reflect a common neurodevelopmental vulnerability of a shared diathesis involving disrupted fronto-striatal and limbic circuitry [70]. Clinically, the presence of these additional comorbidities suggests that BD-OCD patients represent a high-risk subgroup in need of integrated, multimodal treatment approaches.

The increased risk of suicide attempts among adults with BD-OCD represents perhaps our most clinically concerning finding. BD already carries a high suicide risk [71], and our analysis suggests that OCD comorbidity may increase this risk further through multiple mechanisms. The combination of impulsivity from BD with the intense distress and rumination characteristic of OCD may create a particularly dangerous clinical picture [72]. Additionally, the treatment complexity and potentially poorer response to standard interventions in the comorbid group [44] may contribute to hopelessness and heightened suicide risk. Moreover, the increased functional impairment we observed in this population likely contributes to reduced quality of life, exacerbating suicidal tendencies.

This significantly reduced functioning in adults with BD-OCD reflects the cumulative impact of managing two severe mental health conditions simultaneously. Both BD and OCD independently may cause substantial functional impairment through different mechanisms, BD through episodic mood disturbances and cognitive impairment [73, 74], and OCD through time-consuming rituals and pervasive avoidance behaviors [75]. When combined, these conditions appear to create a synergistic negative impact on functioning that supports the need for integrated treatment approaches specifically targeting functional recovery alongside symptom reduction in this population.

Another finding that may help explain these poor outcomes is the earlier age at onset of BD in those with comorbid OCD. Earlier onset of BD is generally associated with a longer delay to treatment or more severe depression [76]. The earlier onset suggests that OCD comorbidity may be a marker of a more developmentally pervasive form of BD with stronger neurodevelopmental underpinnings. The longer lifetime exposure to illness burden resulting from this earlier onset may partially explain both the reduced functioning and increased suicide risk observed, creating a concerning clinical trajectory that requires early identification and intervention. However, it is also possible to interpret these data as suggesting that individuals already diagnosed with OCD may receive an earlier diagnosis of BD due to their existing contact with mental health services, which increases the likelihood of recognition when mood symptoms emerge.

To the best of our knowledge, this is the first systematic review with meta-analysis examining the clinical impact of OCD comorbidity in BD. In our previous study [18], we found that individuals with BD-OCD had a distinct obsessive-compulsive symptom profile, characterized by more sexual obsessions and fewer contamination obsessions compared to those with OCD alone. Building on this distinctive symptom profile, our current findings further clarify the clinical relevance of OCD comorbidity in BD, highlighting its association with a more severe and functionally impairing illness course. These results have significant translational value. We identified the clinical aspects most strongly associated with BD-OCD comorbidity, underscoring the elements that clinicians should investigate when evaluating patients with BD and suspected OCD comorbidity to support their clinical assessment. By recognizing these characteristics and providing more accurate diagnoses, we can aim for better management and treatment of these individuals, offering personalized care tailored to their specific situations. Although OCD is a disorder of significant interest with onset often occurring in childhood and adolescence, most of our findings relate to adult populations, and we were unable to identify significant differences in children and adolescents. Therefore, we emphasize the need for future studies to determine whether our observations in adults can also apply to younger populations or whether these represent a different phenotype. More broadly, BD-OCD may represent a transdiagnostic phenotype marked by early-onset illness, poor functional outcomes, and greater suicidality, features that require specialized assessment tools, early intervention strategies, and integrated pharmacological and psychotherapeutic approaches.

The present work has some limitations. First, the majority of the included studies are cross-sectional, which precludes speculation about causal effects or whether BD emerged before OCD or vice versa [77]. Future longitudinal studies may be implemented to address the role of diagnostic hierarchy and to observe the changes of symptomological aspects over time. Second, many of our meta-analyses were based on only two studies, and sample sizes were in some cases small. This raises concerns regarding statistical power and the generalizability of our results [78]. Given the clinical importance of this topic, future studies are encouraged to improve our knowledge in this area. Third, due to the limited number of studies available, we were unable to control our results for potential confounding variables such as demographics, mood state, BD type, or medication regimens [79] through meta-regression analyses. For the few comparisons that included at least ten studies, we conducted meta-regressions but did not find any significant effects of the variables examined. Fourth, the onset sequence was reported in fewer than half of the studies, with detailed information available in only about a quarter of them. The onset sequence appears to be highly heterogeneous within this comorbid population [9]; therefore, future studies should report this information as comprehensively as possible to improve diagnostic accuracy and inform therapeutic approaches. Fifth, we found that individuals with BD-OCD have an increased likelihood of comorbid eating disorders. However, eating disorders include multiple conditions characterized by distinct psychopathology, clinical course, and treatment profiles. Given that the original studies reported eating disorders as a unified category, our analysis reflects this grouping, although it may limit detailed insights into their specific impact on BD-OCD. Future studies should aim to report comorbidities with greater specificity, enabling more granular and precise analyses. Sixth, although we included only studies that diagnosed psychiatric conditions using validated diagnostic criteria, different versions of the same criteria may influence diagnostic outcomes. For example, changes such as the required level of insight and the reclassification of OCD outside the anxiety disorders category in DSM-5 may have affected how OCD diagnosis was operationalized across studies. Seventh, we were unable to assess publication bias for the majority of the comparisons due to the requirement of at least ten studies, and we found evidence of publication bias regarding one comparison. Nevertheless, we conducted a comprehensive search strategy across four major databases without time, age, or language restrictions, thereby enhancing our chances of retrieving all relevant data and suggesting minimal publication bias overall.

In conclusion, our meta-analysis indicates that individuals with comorbid BD-OCD present a distinct and more severe clinical profile compared to those with BD alone. These patients demonstrate earlier illness onset, more complex illness course, higher rates of specific psychiatric comorbidities, increased suicide risk, and greater functional impairment. Clinically, these features suggest the importance of recognizing OCD comorbidity as a potential marker of greater illness complexity, guiding clinicians toward more cautious, mood-centered treatment approaches. Further research, and particularly clinical trials, are needed to provide better guidance on treatment-related benefits and risks in this population. The role of antidepressants with anti-OCD properties and their potential impact on the BD illness course and rapid cycling needs to be ascertained. Integrating this perspective into clinical practice may help reduce unnecessary pharmacological burden, improve functional outcomes, and ultimately lead to more personalized care for patients with BD.

## Supporting information

10.1192/j.eurpsy.2025.10087.sm001 De Prisco et al. supplementary material De Prisco et al. supplementary material

## Data Availability

The datasets used for this research are available on request.

## Supplementary material

The supplementary material for this article can be found at http://doi.org/10.1192/j.eurpsy.2025.10087.
