# Supplementary material for: Clinical impact of obsessive-compulsive disorder comorbidity in bipolar disorder: a systematic review and meta-analysis: OCD in BD: A Review on Clinical Impact
Source: Eur Psychiatry. 2025 Aug 26;68(1):e142. doi: 10.1192/j.eurpsy.2025.10087 (PMC12646178; doi:10.1192/j.eurpsy.2025.10087)
Supplement: De Prisco et al. supplementary material [file S0924933825100874sup001.docx]

**Clinical impact of obsessive-compulsive disorder comorbidity in bipolar disorder: a systematic review and meta-analysis**

Michele De Prisco ^1,2,3,4,ψ^, Cristiana Tapoi ^5,ψ^, Vincenzo Oliva ^1,2,3,4^, Robertas Strumila ^6,7,8^, Christine Takami ^9^, Nicolaja Girone ^10^, Monica Macellaro ^10^, Juliana Braga de Salles Andrade ^11^, Christian Nikolaus Schmitz ^12,13,14^, Eduard Vieta ^1,2,3,4*^, Giovanna Fico ^1,2,3^

^1^ Institute of Neurosciences (ICN), Department of Medicine, Faculty of Medicine and Health Sciences, Universitat de Barcelona (UB), c. Casanova, 143, 08036 Barcelona, Spain.

^2^ Bipolar and Depressive Disorders Unit, Hospìtal Clinic de Barcelona. c. Villarroel, 170, 08036 Barcelona, Spain.

^3^ Institut d’Investigacions Biomèdiques August Pi i Sunyer (IDIBAPS), c. Villarroel, 170, 08036 Barcelona, Spain.

^4^ Centro de Investigación Biomédica en Red de Salud Mental (CIBERSAM), Instituto de Salud Carlos III, Madrid, Spain

^5^ Prof. Dr. Alexandru Obregia Clinical Psychiatry Hospital, Bucharest, Romania.

^6^ Department of Urgent and Post Urgent Psychiatry, CHU Montpellier, 34000 Montpellier, France.

^7^ Institute of Functional Genomics, University of Montpellier, CNRS, INSERM, Montpellier, France.

^8^ Faculty of Medicine, Institute of Clinical Medicine, Psychiatric Clinic, Vilnius University, Vilnius, Lithuania.

^9^ Department of Medical Epidemiology and Biostatistics, Karolinska Institutet, Stockholm, Sweden.

^10^ Department of Biomedical and Clinical Sciences “Luigi Sacco”, Department of Psychiatry, University of Milan, Milan, Italy.

^11^ D'Or Institute for Research and Education - IDOR, Rio de Janeiro, Brazil.

^12^ Department of Molecular Neuroimaging, Central Institute of Mental Health, Medical Faculty Mannheim, Heidelberg University, Mannheim, Germany.

^13^ Department of Psychiatry and Psychotherapy, Central Institute of Mental Health, Medical Faculty Mannheim, Heidelberg University, Mannheim, Germany.

^14^ German Center for Mental Health (DZPG), partner site Mannheim.

ψ The authors contributed equally

* Corresponding Author

Prof. Eduard Vieta, MD, PhD

Bipolar and Depressive Disorders Unit,

Institute of Neuroscience

IDIBAPS CIBERSAM

Hospital Clinic, University of Barcelona,

170 Villarroel St 12-0, 08036 Barcelona, Catalonia, Spain.

Email: [evieta@clinic.cat](mailto:evieta@clinic.cat)

**Supplementary Materials**

[Abbreviations used in the Supplementary Materials 3](#_Toc203468884)

[Appendix I 4](#_Toc203468885)

[Deviations from the original protocol 4](#_Toc203468886)

[Appendix II 5](#_Toc203468887)

[Search Strategy 5](#_Toc203468888)

[eTable n.1 - Excluded studies with reasons 6](#_Toc203468889)

[Appendix IV 15](#_Toc203468890)

[eTable n.2 – Onset sequence in people diagnosed with BD-OCD 15](#_Toc203468891)

[eTable n.3 - Quality assessment of the included studies, cross-sectional 16](#_Toc203468892)

[eTable n.4 - Quality assessment of the included studies, longitudinal 17](#_Toc203468893)

[Appendix V 18](#_Toc203468894)

[Main analysis 18](#_Toc203468895)

[eTable n.5 - Meta-regression analyses 19](#_Toc203468896)

[eTable n.6 - Sensitivity analyses, leave-one-out sensitivity analysis 20](#_Toc203468897)

[eTable n.7 - Sensitivity analyses, considering only good quality studies 24](#_Toc203468898)

[Publication bias 25](#_Toc203468899)

[eTable n.8 – Publication bias 26](#_Toc203468900)

[Appendix VI 27](#_Toc203468901)

[eTable 9 - PRISMA Checklist 27](#_Toc203468902)

# Abbreviations used in the Supplementary Materials

*AHRQ - Agency for Healthcare Research and Quality*

*BD - Bipolar Disorder*

*CI - Confidence Interval*

*OCD - Obsessive-Compulsive Disorder*

*OR - Odds Ratio*

*PRISMA - Preferred Reporting Items for Systematic Reviews and Meta-Analyses*

*SMD - Standardized Mean Difference*

# Appendix I

## Deviations from the original protocol

In our original protocol, we planned to conduct meta-regressions for comparisons with high heterogeneity, defined as a Cochran’s Q test p-value <0.10 or an I^2^ statistic >50%, regardless of the number of included studies. However, we revised our approach and opted to perform meta-regressions only for comparisons that included at least ten studies reporting on the specific variable, as recommended in the Cochrane Handbook (Section 10.11.4).

# Appendix II

## Search Strategy

Pubmed/MEDLINE (inception to 15/04/2024: 1,663 results)

("bipolar disorder"[Title/Abstract] OR "mania"[Title/Abstract] OR "hypomania"[Title/Abstract] OR "manic"[Title/Abstract] OR "hypomanic"[Title/Abstract] OR "bipolar"[Title/Abstract] OR "bipolar depression"[Title/Abstract] OR "euthymia"[Title/Abstract] OR "euthymic"[Title/Abstract] OR "mixed features"[Title/Abstract] OR "bipolar disorder"[MeSH Terms]) AND ("obsessive compulsive disorder"[Title/Abstract] OR "ocd"[Title/Abstract] OR "obsessive"[Title/Abstract] OR "obsession"[Title/Abstract] OR "compulsive"[Title/Abstract] OR "compulsion"[Title/Abstract] OR "obsessive compulsive disorder"[MeSH Major Topic])

Scopus (inception to 15/04/2024: 4,688 results)

(TITLE-ABS("bipolar disorder") OR TITLE-ABS(mania) OR TITLE-ABS(hypomania) OR TITLE-ABS(manic) OR TITLE-ABS(hypomanic) OR TITLE-ABS(bipolar) OR TITLE-ABS("bipolar depression") OR TITLE-ABS(euthymia) OR TITLE-ABS(euthymic) OR TITLE-ABS("mixed features") OR INDEXTERMS("bipolar disorder")) AND (TITLE-ABS("obsessive compulsive disorder") OR TITLE-ABS(ocd) OR TITLE-ABS(obsessive) OR TITLE-ABS(obsession) OR TITLE-ABS(compulsive) OR TITLE-ABS(compulsion) OR INDEXTERMS("obsessive compulsive disorder"))

PsycINFO (inception to 15/04/2024: 1,417 results)

((TI "bipolar disorder" OR AB "bipolar disorder") OR (TI mania OR AB mania) OR (TI hypomania OR AB hypomania) OR (TI manic OR AB manic) OR (TI hypomanic OR AB hypomanic) OR (TI bipolar OR AB bipolar) OR (TI "bipolar depression" OR AB "bipolar depression") OR (TI euthymia OR AB euthymia) OR (TI euthymic OR AB euthymic) OR (TI "mixed features" OR AB "mixed features") OR (MH "bipolar disorder+")) AND ((TI "obsessive compulsive disorder" OR AB "obsessive compulsive disorder") OR (TI ocd OR AB ocd) OR (TI obsessive OR AB obsessive) OR (TI obsession OR AB obsession) OR (TI compulsive OR AB compulsive) OR (TI compulsion OR AB compulsion) OR (MM "obsessive compulsive disorder+"))

WoS (inception to 15/04/2024: 4,191 results)

((TI="bipolar disorder" OR AB="bipolar disorder") OR (TI=mania OR AB=mania) OR (TI=hypomania OR AB=hypomania) OR (TI=manic OR AB=manic) OR (TI=hypomanic OR AB=hypomanic) OR (TI=bipolar OR AB=bipolar) OR (TI="bipolar depression" OR AB="bipolar depression") OR (TI=euthymia OR AB=euthymia) OR (TI=euthymic OR AB=euthymic) OR (TI="mixed features" OR AB="mixed features")) AND ((TI="obsessive compulsive disorder" OR AB="obsessive compulsive disorder") OR (TI=ocd OR AB=ocd) OR (TI=obsessive OR AB=obsessive) OR (TI=obsession OR AB=obsession) OR (TI=compulsive OR AB=compulsive) OR (TI=compulsion OR AB=compulsion))

**Appendix III**

## eTable n.1 - Excluded studies with reasons

| **Author, year** | **Reason for exclusion** |
| --- | --- |
| Abdel Hamid et al., 2019 | Wrong population |
| Ahn-Robbins et al., 2022 | Wrong population |
| Akiskal et al., 2006 | Wrong population |
| Albert et al., 2012 | Wrong population |
| Almeida Barradas et al., 2020 | Wrong publication type |
| Altinbas et al., 2021 | Wrong publication type |
| Altindag et al., 2006 | Wrong population |
| Angst et al., 2005 | Wrong population |
| Baldessarini et al., 2008 | Wrong outcome |
| Baptista et al., 2020 | Wrong population |
| Baxter et al., 1989 | Wrong population |
| Benatti et al., 2014 | Wrong population |
| Berkol et al., 2021 | Wrong population |
| Bogetto et al., 2007 | Wrong publication type |
| Braverman et al., 2021 | Wrong population |
| Cavicchioli et al., 2018 | Wrong population |
| Cederlof et al., 2015 | Wrong population |
| Chen et al., 1995 | Duplicate |
| Chen et al., 2021 | Wrong outcome |
| Cho et al., 2024 | Wrong population |
| Corlay et al., 2018 | Wrong publication type |
| Cosoff et al., 1998 | Wrong population |
| Dell'Osso et al., 2011 | Unrelated to our research question |
| Dhar et al., 2015 | Wrong publication type |
| Dilsaver et al., 2008 | Wrong population |
| Fagiolini et al., 2007 | Unrelated to our research question |
| Ferrao et al., 2023 | Wrong publication type |
| Fornaro et al., 2021 | Wrong population |
| Galimberti et al., 2020 | Wrong population |
| Gao et al., 2008 | Unrelated to our research question |
| Grillault Laroche et al., 2022 | Unrelated to our research question |
| Henry et al., 2003 | Wrong population |
| Issler et al., 2005 | Unrelated to our research question |
| Jeon et al., 2017 | Wrong publication type |
| Joshi et al., 2006 | Wrong publication type |
| Joshi et al., 2010 | Overlapping sample |
| Keck et al., 2007 | Wrong publication type |
| Keskin et al., 2014 | Wrong publication type |
| Khafif et al., 2021 | Unrelated to our research question |
| Kinrys et al., 2019 | Wrong population |
| Lakhal et al., 2008 | Wrong publication type |
| Lane et al., 2006 | Unrelated to our research question |
| Lee et al., 2008 | Wrong population |
| Lee et al., 2022 | Unrelated to our research question |
| Lucassen et al., 2022 | Wrong publication type |
| Maina et al., 2018 | Wrong publication type |
| Martiadis et al., 2023 | Wrong publication type |
| Masi et al., 2013 | Wrong population |
| McKay et al., 1994 | Wrong publication type |
| Mostafavil et al., 2000 | Wrong publication type |
| Ndetei et al., 2008 | Wrong population |
| Newport et al., 2012 | Wrong population |
| Oedegaard et al., 2008 | Wrong population |
| Oral et al., 2009 | Wrong publication type |
| Pashinian et al., 2006 | Wrong population |
| Patra et al., 2016 | Wrong publication type |
| Paul et al., 2015 | Wrong population |
| Pavlova et al., 2018 | Wrong population |
| Perugi et al., 2001 | Wrong population |
| Pini et al., 1997 | Wrong population |
| Pini et al., 2003 | Wrong population |
| Prisciandaro et al., 2019 | Wrong population |
| Raja et al., 2003 | Wrong publication type |
| Ratheesh et al., 2011 | Wrong population |
| Robbins et al., 2022 | Wrong population |
| Robins et al., 2022 | Wrong population |
| Rock et al., 2013 | Wrong population |
| Rowe et al., 2023 | Wrong population |
| Salvo et al., 2021 | Wrong population |
| Saunders et al., 2012 | Wrong population |
| Shabani et al., 2008 | Wrong population |
| Sharma et al., 2008 | Unrelated to our research question |
| Sharma et al., 2018 | Wrong publication type |
| Sharma et al., 2019 | Wrong publication type |
| Shon et al., 2014 | Wrong publication type |
| Si et al., 2008 | Wrong population |
| Souery et al., 2011 | Wrong population |
| Strakowski et al., 1998 | Unrelated to our research question |
| Tamam et al., 2002 | Authors contacted Sep 17th |
| Teh et al., 2020 | Not diagnosed according DSM/ICD criteria |
| Tonna et al., 2015 | Wrong publication type |
| Tonna et al., 2015 | Wrong publication type |
| Tonna et al., 2016 | Wrong publication type |
| Tonna et al., 2021 | Unrelated to our research question |
| Vaschetto et al., 1996 | Unrelated to our research question |
| Verdolini et al., 2014 | Wrong population |
| Vilela et al., 2014 | Wrong publication type |
| Zerdzinski et al., 2016 | Wrong population |
| Zutshi et al., 2006 | Unrelated to our research question |
| Zutshi et al., 2007 | Wrong population |

**References**

Abdel Hamid, A.A.L., Nasreldin, M., Gohar, S.M., Saleh, A.A., Tarek, M.-A., 2019. Sexual and Religious Obsessions in Relation to Suicidal Ideation in Bipolar Disorder. Suicide and Life-Threatening Behavior 49, 1552-1559.

Ahn-Robbins, D., Grootendorst-van Mil, N.H., Chang, C.-K., Chandran, D., Shetty, H., Sanyal, J., MacCabe, J.H., Cohen, H., Stewart, R., Schirmbeck, F., de Haan, L., Hayes, R.D., 2022. Prevalence and Correlates of Obsessive-Compulsive Symptoms in Individuals With Schizophrenia, Schizoaffective Disorder, or Bipolar Disorder. The Journal of clinical psychiatry 83.

Akiskal, H.S., Akiskal, K.K., Perugi, G., Toni, C., Ruffolo, G., Tusini, G., 2006. Bipolar II and anxious reactive "comorbidity": Toward better phenotypic characterization suitable for genotyping. Journal of Affective Disorders 96, 239-247.

Albert, U., Aguglia, A., Barbaro, F., De Cori, D., Maina, G., Bogetto, F., 2012. Gender related differences in obsessive-compulsive disorder: A clinical study on a sample of 415 patients. Quaderni Italiani di Psichiatria 31, 190-200.

Almeida Barradas, N., Delgado, R.M., Marques, C., 2020. The clinical and therapeutic challenges of comorbid obsessive-compulsive disorder and bipolar disorder in children and adolescents. European Neuropsychopharmacology 40, S405-S405.

Altinbas, K., 2021. Treatment of Comorbid Psychiatric Disorders with Bipolar Disorder. Noropsikiyatri Arsivi-Archives of Neuropsychiatry 58, S41-S46.

Altindag, A., Yanik, M., Nebioglu, M., 2006. The comorbidity of anxiety disorders in bipolar I patients: Prevalence and clinical correlates. Israel Journal of Psychiatry and Related Sciences 43, 10-15.

Angst, J., Gamma, A., Endrass, J., Hantouche, E., Goodwin, R., Ajdacic, V., Eich, D., Rössler, W., 2005. Obsessive-compulsive syndromes and disorders -: Significance of comorbidity with bipolar and anxiety syndromes. European Archives of Psychiatry and Clinical Neuroscience 255, 65-71.

Baldessarini, R.J., Perry, R., Pike, J., 2008. Factors associated with treatment nonadherence among US bipolar disorder patients. Human Psychopharmacology-Clinical and Experimental 23, 95-105.

Baptista, T., Galue, L., Martinez, F., 2020. Bidirectional comorbidity between bipolar- and obsessive-compulsive disorders: symptoms frequency, treatment challenges and underexplored areas. Investigacion Clinica 61, 189-195.

Baxter, L.R., Jr., Schwartz, J.M., Phelps, M.E., Mazziotta, J.C., Guze, B.H., Selin, C.E., Gerner, R.H., Sumida, R.M., 1989. Reduction of prefrontal cortex glucose metabolism common to three types of depression. Arch Gen Psychiatry 46, 243-250.

Benatti, B., Dell'Osso, B., Arici, C., Hollander, E., Altamura, A.C., 2014. Characterizing impulsivity profile in patients with obsessive-compulsive disorder. International Journal of Psychiatry in Clinical Practice 18, 156-160.

Berkol, T.D., Aytac, H.M., 2021. Comparison of Clinical Features of Bipolar Disorder Patients with and without Psychiatric Comorbidity. Eurasian Journal of Medicine 53, 203-207.

Bogetto, F., Asinari, G.F., Pessina, E., Maina, G., 2007. Comorbidity in bipolar and anxiety disorders. Minerva Psichiatrica 48, 55-73.

Braverman, L., Fuchs, C., Weizman, A., Poyurovsky, M., 2021. Elevated rate of OCD-spectrum and tic disorders in patients with bipolar depression and comorbid OCD. Journal of Obsessive-Compulsive and Related Disorders 29.

Cavicchioli, F.L., Maes, M., Roomruangwong, C., Bonifacio, K.L., Barbosa, D.S., Anderson, G., Vargas, H.O., Vargas Nunes, S.O., 2018. Associations between severity of anxiety and clinical and biological features of major affective disorders. Psychiatry Research 260, 17-23.

Cederlof, M., Lichtenstein, P., Larsson, H., Boman, M., Ruck, C., Landen, M., Mataix-Cols, D., 2015. Obsessive-Compulsive Disorder, Psychosis, and Bipolarity: A Longitudinal Cohort and Multigenerational Family Study. Schizophrenia Bulletin 41, 1076-1083.

Chen, C., Chan, H.Y., Yeh, L.L., Pan, Y.J., 2021. Longitudinal factors associated with mortality in older patients with mood disorders. Journal of Affective Disorders 278.

Chen, Y.W., Dilsaver, S.C., 1995. Comorbidity for obsessive-compulsive disorder in bipolar and unipolar disorders. Psychiatry Research 59, 57-64.

Cho, C.H., Son, S., Lee, Y., Jeong, J., Yeom, J.W., Seo, J.Y., Moon, E., Baek, J.H., Park, D.Y., Kim, S.J., Ha, T.H., Cha, B., Kang, H.J., Ahn, Y.M., An, H., Lee, H.J., 2024. Identifying predictive factors for mood recurrence in early-onset major mood disorders: A 4-year, multicenter, prospective cohort study. Psychiatry Research 335.

Corlay, I., Nicolini, H., Islas, V., Medahua, S., 2018. Bipolar disorder with comorbid obsessive compulsive disorder. One treatment uncovers the other. Bipolar Disorders 20, 85-85.

Cosoff, S.J., Hafner, R.J., 1998. The prevalence of comorbid anxiety in schizophrenia, schizoaffective disorder and bipolar disorder. Australian and New Zealand Journal of Psychiatry 32, 67-72.

Dell'Osso, B., Buoli, M., Bortolussi, S., Camuri, G., Vecchi, V., Altamura, A.C., 2011. Patterns of Axis I comorbidity in relation to age in patients with Bipolar Disorder: A cross-sectional analysis. Journal of Affective Disorders 130, 318-322.

Dhar, P., 2015. A study to assess disability among patients of bipolar affective disorders & obsessive compulsive disorder. Indian Journal of Psychiatry 57, S47-S47.

Dilsaver, S.C., Benazzi, F., Akiskal, K.K., Akiskal, H.S., 2008. Differential patterns of lifetime multiple anxiety disorder comorbidity between Latino adults with bipolar I and major depressive disorders. Bulletin of the Menninger Clinic 72, 130-148.

Fagiolini, A., Frank, E., Rucci, P., Cassano, G.B., Turkin, S., Kupfer, D.J., 2007. Mood and anxiety spectrum as a means to identify clinically relevant subtypes of bipolar I disorder. Bipolar Disorders 9, 462-467.

Ferrao, Y.A., Bertoluci, M., Boff, L.S., Beckhauser, H., Ghiorzi, I., Langa, G., 2023. Bipolar and obsessive-compulsive disorders psychopathological intersection: An exploratory study. European Psychiatry 66, S232-S232.

Fornaro, M., Novello, S., Fusco, A., Anastasia, A., De Prisco, M., Mondin, A.M., Mosca, P., Iasevoli, F., de Bartolomeis, A., 2021. Clinical features associated with early drop-out among outpatients with unipolar and bipolar depression. Journal of Psychiatric Research 136, 522-528.

Galimberti, C., Caricasole, V., Bosi, M.F., Viganò, C.A., Ketter, T.A., Dell'Osso, B., 2020. Clinical features and patterns of psychopharmacological prescription in bipolar patients with vs without anxiety disorders at onset. Early Intervention in Psychiatry 14, 714-722.

Gao, K., Tolliver, B.K., Kemp, D.E., Verduin, M.L., Ganocy, S.J., Bilali, S., Brady, K.T., Shim, S.S., Findling, R.L., Calabrese, J.R., 2008. Differential interactions between, comorbid anxiety disorders and substance use disorder in rapid cycling bipolar I or II disorder. Journal of Affective Disorders 110, 167-173.

Grillault Laroche, D., Godin, O., Dansou, Y., Belzeaux, R., Aouizerate, B., Burté, T., Courtet, P., Dubertret, C., Haffen, E., Llorca, P.M., Olie, E., Roux, P., Polosan, M., Schwan, R., Leboyer, M., Bellivier, F., Marie-Claire, C., Etain, B., 2022. Influence of childhood maltreatment on prevalence, onset, and persistence of psychiatric comorbidities and suicide attempts in bipolar disorders. European Psychiatry 65.

Henry, C., Van den Bulke, D., Bellivier, F., Etain, B., Rouillon, F., Leboyer, M., 2003. Anxiety disorders in 318 bipolar patients: Prevalence and impact on illness severity and response to mood stabilizer. Journal of Clinical Psychiatry 64, 331-335.

Issler, C.K., Amaral, J.A., Tamada, R.S., Schwartzmann, A.M., Shavitt, R.G., Miguel, E.C., Lafer, B., 2005. Clinical expression of obsessive-compulsive disorder in women with bipolar disorder. Braz J Psychiatry 27, 139-142.

Jeon, S., Baek, J.H., Yang, S.Y., Choi, Y., Ahn, S.W., Ha, K., Hong, K.S., 2017. Comorbidity rate, clinical nature, and correlates of obsessive-compulsive disorder in patients with bipolar disorders. Bipolar Disorders 19, 91-91.

Joshi, G., Mick, E., Wozniak, J., Geller, D., Park, J., Strauss, S., Biederman, J., 2010. Impact of obsessive-compulsive disorder on the antimanic response to olanzapine therapy in youth with bipolar disorder. Bipolar Disorders 12, 196-204.

Joshi, G., Wozniak, J., Geller, D., Petty, C., Vivas, F., Biederman, J., 2006. Examining the clinical characteristics by comorbidity status to explore the relationship between comorbid bipolar disorder and obsessive-compulsive disorder in children and adolescents. International Journal of Neuropsychopharmacology 9, S180-S181.

Keck, P.E., Jr., 2007. Co-occurrence of bipolar disorder, obsessive compulsive disorder and impulse control disorders. Bipolar Disorders 9, 4-4.

Keskin, N., Tamam, L., 2014. Bipolar disorder and obsessive compulsive disorder comorbidity. Psikiyatride Guncel Yaklasimlar 6, 429-437.

Khafif, T.C., Belizario, G.O., Silva, M., Gomes, B.C., Lafer, B., 2021. Quality of life and clinical outcomes in bipolar disorder: An 8-year longitudinal study. Journal of Affective Disorders 278, 239-243.

Kinrys, G., Bowden, C.L., Nierenberg, A.A., Hearing, C.M., Gold, A.K., Rabideau, D.J., Sylvia, L.G., Gao, K., Kamali, M., Bobo, W.V., Tohen, M., Deckersbach, T., McElroy, S.L., Ketter, T.A., Shelton, R.C., Friedman, E.S., Calabrese, J.R., McInnis, M.G., Kocsis, J., Thase, M.E., Singh, V., Reilly-Harrington, N.A., 2019. Comorbid anxiety in bipolar CHOICE: Insights from the bipolar inventory of symptoms scale. Journal of Affective Disorders 246, 126-131.

Lakhal, N., Homri, W., Cheour, M., Lakhdhar, M.A.B., El Kefi, H., 2008. Bipolar obsessive-compulsive-disorder. European Psychiatry 23, S352-S352.

Lane, J.C., Doran, C.M., 2006. Alice in Wonderland meets bipolar disorder and OCD: The interface of neurology and psychiatry. Headache and Pain: Diagnostic Challenges, Current Therapy 17, 46-48.

Lee, J.H., Dunner, D.L., 2008. The effect of anxiety disorder comorbidity on treatment resistant bipolar disorders. Depression and Anxiety 25, 91-97.

Lee, Y., Lee, D., Jung, H., Cho, Y., Baek, J.H., Hong, K.S., 2022. Heterogeneous early illness courses of Korean patients with bipolar disorders: replication of the staging model. Bmc Psychiatry 22.

Lucassen, L., Tioli, I., Ferrari, M., Ossola, P., Marchesi, C., 2022. The role of Executive Attention in the association between obsessive-compulsive symptoms and relapses in Major Depressive and Bipolar Disorder. European Psychiatry 65, S158-S158.

Maina, G., Di Salvo, G., Rosso, G., 2018. Does comorbid obsessive-compulsive disorder influence suicidality in patients with bipolar disorder? Bipolar Disorders 20, 87-87.

Martiadis, V., Pessina, E., Martini, A., Raffone, F., Giunnelli, P., De Berardis, D., 2023. LAI-2 adjunctive treatment for type I Bipolar patients with comorbid Obsessive Compulsive Disorder: preliminary data from a real-world multi-centric Italian clinical experience. European Psychiatry 66, S712-S712.

Masi, G., Pisano, S., Pfanner, C., Milone, A., Manfredi, A., 2013. Quetiapine Monotherapy in adolescents with bipolar disorder Comorbid with conduct disorder. Journal of Child and Adolescent Psychopharmacology 23, 568-571.

McKay, D.R., Yaryuratobias, J.A., Neziroglu, F.A., 1994. OBSESSIVE-COMPULSIVE AND BIPOLAR DISORDER - PRELIMINARY OUTCOME DATA. Biological Psychiatry 35, 738-738.

Mostafavil, H., Aleah, B., 2000. Clonidine in bipolar disorder with OCD. Neuropsychopharmacology 23, S132-S132.

Ndetei, D.M., Pizzo, M., Ongecha, F.A., Khasakhala, L.I., Maru, H., Mutiso, V., Kokonya, D.A., 2008. Obsessive-compulsive (oc) symptoms in psychiatric in-patients at Mathari hospital, Kenya. African Journal of Psychiatry (South Africa) 11, 182-186.

Newport, D.J., Baldessarini, R.J., Knight, B.T., Fernandez, S.V., Morris, N.J., Viguera, A.C., Stowe, Z.N., 2012. Comparison of Women With Confirmed Versus Presumably Misdiagnosed Bipolar Disorder. Journal of Clinical Psychiatry 73, 242-246.

Oedegaard, K.J., Neckelmann, D., Benazzi, F., Syrstad, V.E.G., Akiskal, H.S., Fasmer, O.B., 2008. Dissociative experiences differentiate bipolar-II from unipolar depressed patients: The mediating role of cyclothymia and the type A behaviour speed and impatience subscale. Psiquiatria Biologica 15, 202-213.

Oral, E., Ozan, E., Deveci, E., Aydin, N., Kirpinar, I., 2009. BIPOLAR DISORDER (BD) AND OBSESSIVE COMPULSIVE DISORDER (OCD) COMORBIDTY OR VARIED EPISODES IN THE SAME DISORDER. European Psychiatry 24.

Pashinian, A., Faragian, S., Levi, A., Yeghiyan, M., Gasparyan, K., Weizman, R., Weizman, A., Fuchs, C., Poyurovsky, M., 2006. Obsessive-compulsive disorder in bipolar disorder patients with first manic episode. Journal of Affective Disorders 94, 151-156.

Patra, S., 2016. Treat the disease not the symptoms: Successful management of obsessive compulsive disorder in bipolar disorder with aripiprazole augmentation. Australian and New Zealand Journal of Psychiatry 50, 809-810.

Paul, I., Sinha, V.K., Sarkhel, S., Praharaj, S.K., 2015. Co-morbidity of Obsessive-compulsive Disorder and Other Anxiety Disorders with Child and Adolescent Mood Disorders. East Asian Arch Psychiatry 25, 58-63.

Pavlova, B., Perroud, N., Cordera, P., Uher, R., Alda, M., Dayer, A., Aubry, J.M., 2018. Anxiety disorders and childhood maltreatment as predictors of outcome in bipolar disorder. J Affect Disord 225, 337-341.

Perugi, G., Frare, F., Toni, C., Mata, B., Akiskal, H.S., 2001. Bipolar II and unipolar comorbidity in 153 outpatients with social phobia. Comprehensive Psychiatry 42, 375-381.

Pini, S., Cassano, G.B., Simonini, E., Savino, M., Russo, A., Montgomery, S.A., 1997. Prevalence of anxiety disorders comorbidity in bipolar depression, unipolar depression and dysthymia. Journal of Affective Disorders 42, 145-153.

Pini, S., Dell'Osso, L., Amador, X.F., Mastrocinoque, C., Saettoni, M., Cassano, G.B., 2003. Awareness of illness in patients with bipolar I disorder with or without comorbid anxiety disorders. Australian and New Zealand Journal of Psychiatry 37, 355-361.

Prisciandaro, J.J., Mellick, W., Mitaro, E., Tolliver, B.K., 2019. An evaluation of the impact of co-occurring anxiety and substance use disorder on bipolar disorder illness outcomes in STEP-BD. Journal of Affective Disorders 246, 794-799.

Raja, M., Azzoni, A., 2003. Obsessive-Compulsive symptoms in mania. European Neuropsychopharmacology 13, S234-S234.

Ratheesh, A., Srinath, S., Reddy, Y.C.J., Girimaji, S., Seshadri, S., Thennarasu, K., Hutin, Y., 2011. Are anxiety disorders associated with a more severe form of bipolar disorder in adolescents. Indian Journal of Psychiatry 53, 312-318.

Robins, D.A., Grootendorst-van Mil, N.H., Chang, C.-K., Chandran, D., Shetty, H., Sanyal, J., MacCabe, J.H., Cohen, H., Stewart, R., Schirmbeck, F., de Haan, L., Hayes, R.D., 2022a. Prevalence and Correlates of Obsessive-Compulsive Symptpms in Individuals With Schizophrenia, Schizoaffective Disorder, or Bipolar Disorder. Journal of Clinical Psychiatry 83.

Robins, D.A., Grootendorst-Van Mil, N.H., Chang, C.K., Chandran, D., Shetty, H., Sanyal, J., MacCabe, J.H., Cohen, H., Stewart, R., Schirmbeck, F., de Haan, L., Hayes, R.D., 2022b. Prevalence and Correlates of Obsessive-Compulsive Symptoms in Individuals with Schizophrenia, Schizoaffective Disorder, or Bipolar Disorder. Journal of Clinical Psychiatry 83.

Rock, P.L., Chandler, R.A., Harmer, C.J., Rogers, R.D., Goodwin, G.M., 2013. The common bipolar phenotype in young people. International Journal of Bipolar Disorders 1, 1-6.

Rowe, A.L., Perich, T., Meade, T., 2023. Cumulative trauma in bipolar disorder: An examination of prevalence and outcomes across the lifespan. Journal of Affective Disorders 327, 254-261.

Salvo, G.D., Maina, G., Pessina, E., Teobaldi, E., Barbaro, F., Martini, A., Albert, U., Rosso, G., 2021. Aripiprazole augmentation to mood stabilizers for obsessive-compulsive symptoms in bipolar disorder. Medicina (Lithuania) 57, 1-8.

Saunders, E.F., Fitzgerald, K.D., Zhang, P., McInnis, M.G., 2012. Clinical features of bipolar disorder comorbid with anxiety disorders differ between men and women. Depress Anxiety 29, 739-746.

Shabani, A., Alizadeh, A., 2008. The specific pattern of obsessive-compulsive symptoms in patients with bipolar disorder. Journal of Research in Medical Sciences 13, 48-54.

Sharma, L.P., Reddy, Y.C.J., 2019. Obsessive-compulsive disorder comorbid with schizophrenia and bipolar disorder. Indian Journal of Psychiatry 61, S140-S148.

Sharma, V., Doobay, M., 2018. Lamotrigine-induced obsessive compulsive disorder in patients with bipolar disorder. Bipolar Disorders 20, 87-88.

Sharma, V., Khan, M., Corpse, C., Sharma, P., 2008. Missed bipolarity and psychiatric comorbidity in women with postpartum depression. Bipolar Disorders 10, 742-747.

Shon, S.H., Joo, Y., Park, J., Youngstrom, E.A., Kim, H.W., 2014. Comparison of clinical characteristics of bipolar and depressive disorders in Korean clinical sample of youth: a retrospective chart review. Eur Child Adolesc Psychiatry 23, 307-316.

Si, T., Shu, L., 2008. Citalopram in the treatment of depressive disorders: an open label, multicenter study in China. African Journal of Pharmacy and Pharmacology 2, 59-65.

Souery, D., Zaninotto, L., Calati, R., Linotte, S., Sentissi, O., Amital, D., Moser, U., Kasper, S., Zohar, J., Mendlewicz, J., Serretti, A., 2011. Phenomenology of psychotic mood disorders: Lifetime and major depressive episode features. Journal of Affective Disorders 135, 241-250.

Strakowski, S.M., Sax, K.W., McElroy, S.L., Keck Jr, P.E., Hawkins, J.M., West, S.A., 1998. Course of psychiatric and substance abuse syndromes co-occurring with bipolar disorder after a first psychiatric hospitalization. Journal of Clinical Psychiatry 59, 465-471.

Tamam, L., Ozpoyraz, N., 2002. Comorbidity of anxiety disorder among patients with bipolar I disorder in remission. Psychopathology 35, 203-209.

Teh, W.L., Abdin, E., Vaingankar, J., Shafie, S., Yiang Chua, B., Sambasivam, R., Zhang, Y., Shahwan, S., Chang, S., Mok, Y.M., Verma, S., Heng, D., Subramaniam, M., Chong, S.A., 2020. Prevalence and correlates of bipolar spectrum disorders in Singapore: Results from the 2016 Singapore Mental Health Study (SMHS 2016). J Affect Disord 274, 339-346.

Tonna, M., Amerio, A., Odone, A., Stubbs, B., Ghaemi, S.N., 2015a. Comorbid bipolar disorder and obsessive-compulsive disorder:state of the art in pediatric patients. Shanghai archives of psychiatry 27, 386-387.

Tonna, M., Amerio, A., Odone, A., Stubbs, B., Ghaemi, S.N., 2016. Comorbid bipolar disorder and obsessive-compulsive disorder: Which came first? Australian and New Zealand Journal of Psychiatry 50, 695-698.

Tonna, M., Amerio, A., Stubbs, B., Odone, A., Ghaemi, S.N., 2015b. Comorbid bipolar disorder and obsessive-compulsive disorder: A child and adolescent perspective. Australian and New Zealand Journal of Psychiatry 49, 1066-1067.

Tonna, M., Trinchieri, M., Lucarini, V., Ferrari, M., Ballerini, M., Ossola, P., De Panfilis, C., Marchesi, C., 2021. Pattern of occurrence of obsessive-compulsive symptoms in bipolar disorder. Psychiatry Research 297.

Vaschetto, P., Bogetto, F., Maina, G., Manfredi, A., Ravizza, L., 1996. La comorbidità tra i disturbi d'ansia ed i disturbi dell'umore = The comorbidity between anxiety and mood disorders. Minerva Psichiatrica 37, 127-134.

Verdolini, N., Dean, J., Elisei, S., Quartesan, R., Zaman, R., Agius, M., 2014. Bipolar disorder: The importance of clinical assessment in identifying prognostic factors - An Audit. Part 2: Mixed state features and rapid cycling. Psychiatria Danubina 26, 301-308.

Vilela, A.C., Azevedo, P.V., Caixeta, L.F., Taveira, D.L., 2014. Trichotillomania associated with bipolar disorder and obsessive compulsive disorder: pathoplasty or comorbidity? Int J Trichology 6, 36-37.

Zerdziński, M., 2016. Diagnosis of obsessive-compulsive disorder in the course of bipolar disorder. Psychiatria i Psychologia Kliniczna 16, 61-67.

Zutshi, A., Kamath, P., Reddy, Y.C.J., 2007. Bipolar and nonbipolar obsessive-compulsive disorder: a clinical exploration. Comprehensive Psychiatry 48, 245-251.

Zutshi, A., Reddy, Y.C., Thennarasu, K., Chandrashekhar, C.R., 2006. Comorbidity of anxiety disorders in patients with remitted bipolar disorder. Eur Arch Psychiatry Clin Neurosci 256, 428-436.

# Appendix IV

## eTable n.2 – Onset sequence in people diagnosed with BD-OCD

The rows are highlighted in green when the study provided detailed information regarding the onset sequence in people diagnosed with BD-OCD.

The rows are highlighted in yellow when the study provided only partial details regarding the onset sequence in people diagnosed with BD-OCD.

| **Author, year** | **BD-OCD, n** | **BD before OCD** | **BD after OCD** | **BD together with OCD** |
| --- | --- | --- | --- | --- |
| Bener et al, 2016 | 92 | Not reported | | |
| Braveman et al, 2021 | 19 | Not reported; however, they reported the mean age at onset of OCD (15.3 ± 3.3) and of BD (25.7 ± 10.9) | | |
| Centorrino et al, 2006 | 16 | Not reported | | |
| Chen et al, 1995 | 35 | Not reported; however, they reported the mean age at onset of OCD (21.6 ± 12.2) and of BD (20.6 ± 10.9) | | |
| De Filippis et al., 2018 | 26 | 13 (50%) | 13 (50%) | 0 (0%) |
| Di Salvo et al., 2020 | 201 | Not reported | | |
| Dilsaver et al, 2006 | 54 | Not reported | | |
| Ezzat et al., 2023 | 26 | Not reported | | |
| Goes et al, 2012 | 89 | Not reported | | |
| Goodwin et al, 2002 | 6 | Not reported | | |
| Hassani et al, 2006 | 25 | Not reported | | |
| Issler et al, 2010 | 15 | Not reported; however, they reported the mean age at onset of OCD (11.6 ± 7.3) and of BD (19 ± 7.2) | | |
| Jeon et al, 2017 | 26* | 9 (34.6%) | 12 (46.2%) | 5 (19.2%) |
| Joshi et al., 2010 | 17 | 17 (100%) | 0 (0%) | 0 (0%) |
| Kazhungil et al., 2017 | 32 | Not reported; however, they reported the mean age at onset of OCD (26.4 ± 10.2) and of BD (24.7 ± 8.4) | | |
| Khan et al, 2019 | 35 | 29 (82.8%) | 6 (17.2%) | 0 (0%) |
| Kim et al, 2014 | 24 | Not reported | | |
| Kocabas et al, 2019 | 28 | Not reported | | |
| Koyoncu et al., 2010 | 35 | Not reported | | |
| Kruger et al., 2000 | 10 | Not reported | | |
| Levander et al, 2007 | 48 | Not reported | | |
| Magalhaes et al, 2010 | 32 | Not reported | | |
| Masi et al, 2004 | 30 | Not reported; however, they reported the mean age at onset of OCD (8.7 ± 2.6) and of BD (11.5 ± 3.1) | | |
| Masi et al, 2018 | 88 | 52 (59.1%) | 36 (40.9%) | 0 (0%) |
| Ozdemiroglu et al, 2015 | 32 | 32 (100%) | 0 (0%) | 0 (0%) |
| Shashidhara et al, 2015 | 29** | 12 (42%) | 13 (45%) | 4 (13%) |

*the information was available only for a subset of the patients with BD-OCD

**the sequence of onset was unclear in one patient with BD-OCD

## eTable n.3 - Quality assessment of the included studies, cross-sectional

| **Author, year** | **Representativeness of the sample (SELECTION)** | **Sample Size (SELECTION)** | **Non-respondents (SELECTION)** | **Ascertainment of the exposure (SELECTION)** | **Comparability (COMPARABILITY)** | **Assessment of the outcome (OUTCOME)** | **Statistical Test (OUTCOME)** | **TOTAL** | **AHRQ Standards** |
| --- | --- | --- | --- | --- | --- | --- | --- | --- | --- |
| Bener et al., 2016 | 1 | 0 | 1 | 2 | 1 | 1 | 1 | 7 | GOOD |
| Braveman et al., 2021 | 1 | 0 | 1 | 1 | 0 | 1 | 1 | 5 | POOR |
| Chen et al., 1995 | 1 | 0 | 0 | 1 | 0 | 1 | 1 | 4 | POOR |
| de Filippis et al., 2018 | 1 | 0 | 0 | 2 | 2 | 1 | 1 | 7 | GOOD |
| Di Salvo et al., 2020 | 1 | 0 | 0 | 1 | 0 | 1 | 1 | 4 | POOR |
| Dilsaver et al., 2006 | 1 | 0 | 0 | 2 | 0 | 1 | 1 | 5 | POOR |
| Ezzat et al., 2023 | 1 | 0 | 1 | 1 | 0 | 1 | 1 | 5 | POOR |
| Goes et al., 2012 | 1 | 0 | 0 | 2 | 0 | 1 | 1 | 5 | POOR |
| Goodwin et al., 2002 | 1 | 0 | 0 | 2 | 0 | 1 | 1 | 5 | POOR |
| Hassani et al., 2006 | 1 | 0 | 0 | 2 | 1 | 1 | 1 | 6 | GOOD |
| Issler et al., 2010 | 1 | 0 | 0 | 2 | 2 | 1 | 1 | 7 | GOOD |
| Jeon et al., 2017 | 1 | 0 | 0 | 2 | 0 | 1 | 1 | 5 | POOR |
| Joshi et al., 2010 | 1 | 0 | 0 | 2 | 0 | 1 | 1 | 5 | POOR |
| Kazhungil et al., 2017 | 1 | 0 | 1 | 2 | 2 | 1 | 1 | 8 | GOOD |
| Khan et al., 2019 | 1 | 0 | 1 | 1 | 2 | 1 | 1 | 7 | GOOD |
| Kocabas et al., 2019 | 1 | 0 | 1 | 2 | 2 | 1 | 1 | 8 | GOOD |
| Koyoncu et al., 2010 | 1 | 0 | 1 | 2 | 2 | 1 | 1 | 8 | GOOD |
| Kruger et al., 2000 | 1 | 0 | 1 | 2 | 0 | 1 | 1 | 6 | POOR |
| Levander et al., 2007 | 1 | 0 | 0 | 2 | 0 | 1 | 1 | 5 | POOR |
| Magalhaes et al., 2010 | 1 | 0 | 0 | 2 | 0 | 1 | 1 | 5 | POOR |
| Ozdemiroglu et al., 2015 | 1 | 0 | 0 | 2 | 2 | 1 | 1 | 7 | GOOD |
| Shashidhara et al., 2015 | 1 | 0 | 1 | 2 | 2 | 1 | 1 | 8 | GOOD |

## eTable n.4 - Quality assessment of the included studies, longitudinal

| **Author, year** | **Representativeness of the exposed cohort (SELECTION)** | **Representativeness of the non-exposed cohort (SELECTION)** | **Ascertainment of the exposure (SELECTION)** | **Outcome of interest was not present at the beginning (SELECTION)** | **Comparability (COMPARABILITY)** | **Assessment of the outcome (OUTCOME)** | **Follow-up long enough (OUTCOME)** | **Adequacy of follow-up (OUTCOME)** | **TOTAL** | **AHRQ Standards** |
| --- | --- | --- | --- | --- | --- | --- | --- | --- | --- | --- |
| Centorrino et al., 2006 | 1 | 1 | 0 | 0 | 2 | 1 | 1 | 1 | 7 | FAIR |
| Kim et al., 2014 | 1 | 1 | 1 | 0 | 1 | 1 | 1 | 1 | 7 | GOOD |
| Masi et al., 2004 | 1 | 1 | 1 | 0 | 2 | 1 | 1 | 1 | 8 | GOOD |
| Masi et al., 2018 | 1 | 1 | 0 | 0 | 0 | 1 | 1 | 1 | 5 | POOR |

# Appendix V

## Main analysis

The forest plots for each meta-analysis are available at https://osf.io/n6jt5/?view_only=7a6c826cd9de4fb396db3ed681dfd5dc.

## eTable n.5 - Meta-regression analyses

| **Age class** | **Outcome type** | **Effect size, type** | **Predictor** | **Studies, n** | **Beta** | **95% CIs** | **p-value** |
| --- | --- | --- | --- | --- | --- | --- | --- |
| Adults | Age at Onset, BD | SMD | Mean age | 11 | 0.001 | -0.072, 0.073 | 0.99 |
| Adults | Age at Onset, BD | SMD | Percentage of females | 11 | -1.293 | -2.607, 0.021 | 0.05 |
| Adults | Suicide, Attempt | OR | Mean age | 10 | 0.088 | -0.029, 0.206 | 0.14 |
| Adults | Suicide, Attempt | OR | Percentage of females | 10 | -0.578 | -3.148, 1.993 | 0.66 |

## eTable n.6 - Sensitivity analyses, leave-one-out sensitivity analysis

The results are highlighted in green when the removal of that particular study changes a previously non-significant association to significant.

The results are highlighted in red when the removal of that particular study changes a previously significant association to not significant.

| **Outcome type** | **Effect size, type** | **Author, year of the study removed** | **Effect size** | **95% CIs** | **p-value** | **I^2^** | **Q test p-value** |
| --- | --- | --- | --- | --- | --- | --- | --- |
| **Adults** |  |  |  |  |  |  |  |
| Affective Episodes, Psychotic Features | OR | Koyoncu et al., 2010 | 0.569 | 0.29, 1.117 | 0.1 | 0 | 0.42 |
| Affective Episodes, Psychotic Features | OR | Issler et al., 2010 | 0.596 | 0.331, 1.073 | 0.08 | 0 | 0.44 |
| Affective Episodes, Psychotic Features | OR | Shashidhara et al., 2015 | 0.847 | 0.38, 1.885 | 0.68 | 0 | 0.8 |
| Affective Episodes, Rapid Cycling | OR | Kruger et al., 2000 | 2.121 | 1.12, 4.015 | 0.021 | 0 | 0.38 |
| Affective Episodes, Rapid Cycling | OR | Koyoncu et al., 2010 | 2.188 | 1.145, 4.179 | 0.018 | 0 | 0.44 |
| Affective Episodes, Rapid Cycling | OR | Magalhaes et al., 2010 | 1.303 | 0.393, 4.319 | 0.66 | 27.21 | 0.28 |
| Affective Episodes, Rapid Cycling | OR | Ozdemiroglu et al., 2015 | 1.384 | 0.515, 3.725 | 0.52 | 30.33 | 0.29 |
| Comorbidity, Alcohol Use Disorder | OR | Chen et al., 1995 | 1.318 | 0.555, 3.124 | 0.53 | 78 | <0.1 |
| Comorbidity, Alcohol Use Disorder | OR | Koyoncu et al., 2010 | 1.156 | 0.527, 2.537 | 0.72 | 80.17 | <0.1 |
| Comorbidity, Alcohol Use Disorder | OR | Goodwin et al., 2002 | 1.181 | 0.558, 2.502 | 0.66 | 79.58 | <0.1 |
| Comorbidity, Alcohol Use Disorder | OR | Goes et al., 2012 | 1.203 | 0.48, 3.016 | 0.69 | 73.15 | <0.1 |
| Comorbidity, Alcohol Use Disorder | OR | Levander et al., 2007 | 1.508 | 0.746, 3.053 | 0.25 | 61.16 | <0.1 |
| Comorbidity, Alcohol Use Disorder | OR | Magalhaes et al., 2010 | 0.853 | 0.522, 1.392 | 0.52 | 40.28 | 0.22 |
| Comorbidity, Generalized Anxiety Disorder | OR | Bener et al., 2016 | 1.719 | 0.815, 3.633 | 0.16 | 0 | 0.83 |
| Comorbidity, Generalized Anxiety Disorder | OR | Kruger et al., 2000 | 1.52 | 0.892, 2.591 | 0.12 | 0 | 0.79 |
| Comorbidity, Generalized Anxiety Disorder | OR | Koyoncu et al., 2010 | 1.454 | 0.865, 2.445 | 0.16 | 0 | 0.86 |
| Comorbidity, Generalized Anxiety Disorder | OR | Di Salvo et al., 2020 | 1.369 | 0.76, 2.467 | 0.3 | 0 | 0.86 |
| Comorbidity, Generalized Anxiety Disorder | OR | Issler et al., 2010 | 1.57 | 0.94, 2.62 | 0.09 | 0 | 0.94 |
| Comorbidity, Generalized Anxiety Disorder | OR | Shashidhara et al., 2015 | 1.516 | 0.907, 2.535 | 0.11 | 0 | 0.79 |
| Comorbidity, Panic Disorder | OR | Bener et al., 2016 | 3.951 | 2.697, 5.795 | <0.001 | 20.63 | 0.51 |
| Comorbidity, Panic Disorder | OR | Jeon et al., 2017 | 3.219 | 1.885, 5.496 | <0.001 | 50.22 | 0.12 |
| Comorbidity, Panic Disorder | OR | Kruger et al., 2000 | 3.442 | 2.206, 5.366 | <0.001 | 43.46 | 0.21 |
| Comorbidity, Panic Disorder | OR | Koyoncu et al., 2010 | 3.245 | 2.028, 5.186 | <0.001 | 47.25 | 0.12 |
| Comorbidity, Panic Disorder | OR | Di Salvo et al., 2020 | 2.787 | 1.95, 3.979 | <0.001 | 1.6 | 0.66 |
| Comorbidity, Panic Disorder | OR | Goes et al., 2012 | 3.241 | 1.765, 5.948 | <0.001 | 40.72 | 0.16 |
| Comorbidity, Panic Disorder | OR | Issler et al., 2010 | 3.228 | 2.022, 5.16 | <0.001 | 47.22 | 0.13 |
| Comorbidity, Panic Disorder | OR | Shashidhara et al., 2015 | 3.277 | 2.073, 5.181 | <0.001 | 46.27 | 0.12 |
| Comorbidity, PTSD | OR | Bener et al., 2016 | 0.677 | 0.118, 3.9 | 0.66 | 0 | 0.83 |
| Comorbidity, PTSD | OR | Koyoncu et al., 2010 | 0.653 | 0.286, 1.492 | 0.31 | 0 | 0.95 |
| Comorbidity, PTSD | OR | Issler et al., 2010 | 0.676 | 0.278, 1.642 | 0.39 | 0 | 0.84 |
| Comorbidity, Social Anxiety Disorder | OR | Bener et al., 2016 | 2.622 | 1.066, 6.456 | 0.036 | 77.57 | <0.1 |
| Comorbidity, Social Anxiety Disorder | OR | Koyoncu et al., 2010 | 2.275 | 0.745, 6.952 | 0.15 | 86 | <0.1 |
| Comorbidity, Social Anxiety Disorder | OR | Di Salvo et al., 2020 | 1.359 | 0.536, 3.449 | 0.52 | 76.25 | <0.1 |
| Comorbidity, Social Anxiety Disorder | OR | Goes et al., 2012 | 1.944 | 0.572, 6.619 | 0.29 | 84.26 | <0.1 |
| Comorbidity, Social Anxiety Disorder | OR | Issler et al., 2010 | 1.906 | 0.599, 6.068 | 0.28 | 88.72 | <0.1 |
| Comorbidity, Social Anxiety Disorder | OR | Shashidhara et al., 2015 | 1.493 | 0.541, 4.121 | 0.44 | 85.07 | <0.1 |
| Comorbidity, Specific Phobia | OR | Bener et al., 2016 | 2.46 | 1.614, 3.743 | <0.001 | 0 | 0.85 |
| Comorbidity, Specific Phobia | OR | Kruger et al., 2000 | 1.483 | 0.453, 4.855 | 0.52 | 71.39 | <0.1 |
| Comorbidity, Specific Phobia | OR | Koyoncu et al., 2010 | 1.649 | 0.525, 5.181 | 0.39 | 73.46 | <0.1 |
| Comorbidity, Specific Phobia | OR | Goes et al., 2012 | 1.401 | 0.39, 5.028 | 0.61 | 61.76 | <0.1 |
| Comorbidity, Specific Phobia | OR | Issler et al., 2010 | 1.336 | 0.467, 3.823 | 0.59 | 66.36 | <0.1 |
| Comorbidity, Substance Use Disorder | OR | Chen et al., 1995 | 1.311 | 0.885, 1.943 | 0.18 | 23.56 | 0.23 |
| Comorbidity, Substance Use Disorder | OR | Jeon et al., 2017 | 1.448 | 1.057, 1.984 | 0.021 | 9.07 | 0.28 |
| Comorbidity, Substance Use Disorder | OR | Kruger et al., 2000 | 1.419 | 1.045, 1.927 | 0.025 | 10.41 | 0.28 |
| Comorbidity, Substance Use Disorder | OR | Koyoncu et al., 2010 | 1.358 | 0.985, 1.872 | 0.06 | 15.11 | 0.28 |
| Comorbidity, Substance Use Disorder | OR | Di Salvo et al., 2020 | 1.194 | 0.82, 1.737 | 0.36 | 0 | 0.39 |
| Comorbidity, Substance Use Disorder | OR | Goodwin et al., 2002 | 1.368 | 0.992, 1.885 | 0.06 | 14.83 | 0.23 |
| Comorbidity, Substance Use Disorder | OR | Goes et al., 2012 | 1.522 | 1.131, 2.05 | 0.006 | 1.64 | 0.3 |
| Comorbidity, Substance Use Disorder | OR | Magalhaes et al., 2010 | 1.279 | 0.888, 1.84 | 0.19 | 19.87 | 0.28 |
| Comorbidity, Substance Use Disorder | OR | Shashidhara et al., 2015 | 1.525 | 1.168, 1.992 | 0.002 | 0 | 0.71 |
| Suicide, Attempt | OR | Centorrino et al., 2006 | 1.857 | 1.178, 2.924 | 0.008 | 73.22 | <0.1 |
| Suicide, Attempt | OR | Chen et al., 1995 | 1.835 | 1.139, 2.956 | 0.013 | 73.17 | <0.1 |
| Suicide, Attempt | OR | Jeon et al., 2017 | 1.968 | 1.232, 3.142 | 0.005 | 71.11 | <0.1 |
| Suicide, Attempt | OR | Kruger et al., 2000 | 1.732 | 1.143, 2.622 | 0.01 | 68.48 | <0.1 |
| Suicide, Attempt | OR | Koyoncu et al., 2010 | 2.01 | 1.31, 3.083 | 0.001 | 68.3 | <0.1 |
| Suicide, Attempt | OR | Di Salvo et al., 2020 | 1.954 | 1.204, 3.171 | 0.007 | 66.83 | <0.1 |
| Suicide, Attempt | OR | Goes et al., 2012 | 1.895 | 1.16, 3.096 | 0.011 | 69.97 | <0.1 |
| Suicide, Attempt | OR | Issler et al., 2010 | 1.895 | 1.203, 2.986 | 0.006 | 73.14 | <0.1 |
| Suicide, Attempt | OR | Kocabas et al., 2019 | 1.974 | 1.25, 3.117 | 0.004 | 71.22 | <0.1 |
| Suicide, Attempt | OR | Hassani et al., 2006 | 1.904 | 1.197, 3.028 | 0.007 | 73.4 | <0.1 |
| Suicide, Attempt | OR | Magalhaes et al., 2010 | 1.48 | 1.201, 1.824 | <0.001 | 0 | 0.25 |
| Suicide, Attempt | OR | Ozdemiroglu et al., 2015 | 1.761 | 1.122, 2.768 | 0.014 | 71.19 | <0.1 |
| Age at Onset, BD | SMD | Bener et al., 2016 | -0.313 | -0.583, -0.044 | 0.023 | 80.73 | <0.1 |
| Age at Onset, BD | SMD | Braveman et al., 2021 | -0.3 | -0.583, -0.016 | 0.038 | 85.65 | <0.1 |
| Age at Onset, BD | SMD | Chen et al., 1995 | -0.3 | -0.592, -0.007 | 0.045 | 85.78 | <0.1 |
| Age at Onset, BD | SMD | Jeon et al., 2017 | -0.254 | -0.549, 0.041 | 0.09 | 85.36 | <0.1 |
| Age at Onset, BD | SMD | Kruger et al., 2000 | -0.295 | -0.579, -0.011 | 0.042 | 85.96 | <0.1 |
| Age at Onset, BD | SMD | Koyoncu et al., 2010 | -0.305 | -0.584, -0.027 | 0.032 | 84.8 | <0.1 |
| Age at Onset, BD | SMD | Kazhungil et al., 2017 | -0.285 | -0.584, 0.014 | 0.06 | 86.77 | <0.1 |
| Age at Onset, BD | SMD | Di Salvo et al., 2020 | -0.253 | -0.551, 0.045 | 0.1 | 82.1 | <0.1 |
| Age at Onset, BD | SMD | Kim et al., 2014 | -0.257 | -0.55, 0.035 | 0.09 | 86.18 | <0.1 |
| Age at Onset, BD | SMD | Issler et al., 2010 | -0.177 | -0.349, -0.006 | 0.042 | 60.78 | <0.1 |
| Age at Onset, BD | SMD | Ozdemiroglu et al., 2015 | -0.252 | -0.54, 0.037 | 0.09 | 85.8 | <0.1 |
| Age at Onset, BD | SMD | Shashidhara et al., 2015 | -0.293 | -0.591, 0.005 | 0.05 | 86.34 | <0.1 |
| Depressive Episodes, Number | SMD | Jeon et al., 2017 | 1.066 | -0.157, 2.289 | 0.09 | 98 | <0.1 |
| Depressive Episodes, Number | SMD | de Filippis et al., 2018 | 0.965 | -0.306, 2.236 | 0.14 | 98.53 | <0.1 |
| Depressive Episodes, Number | SMD | Kazhungil et al., 2017 | 0.96 | -0.315, 2.235 | 0.14 | 98.44 | <0.1 |
| Depressive Episodes, Number | SMD | Goes et al., 2012 | 0.297 | -0.079, 0.674 | 0.12 | 76.17 | <0.1 |
| Depressive Episodes, Number | SMD | Issler et al., 2010 | 0.822 | -0.444, 2.088 | 0.2 | 98.59 | <0.1 |
| Depressive Episodes, Number | SMD | Ozdemiroglu et al., 2015 | 0.984 | -0.282, 2.251 | 0.13 | 98.43 | <0.1 |
| Depressive Episodes, Number | SMD | Shashidhara et al., 2015 | 1.07 | -0.15, 2.289 | 0.09 | 98.19 | <0.1 |
| Duration of Illness, BD | SMD | Bener et al., 2016 | 0.035 | -0.172, 0.242 | 0.74 | 0 | 0.97 |
| Duration of Illness, BD | SMD | Jeon et al., 2017 | -0.193 | -0.515, 0.13 | 0.24 | 56.1 | <0.1 |
| Duration of Illness, BD | SMD | de Filippis et al., 2018 | -0.136 | -0.461, 0.19 | 0.41 | 69.71 | <0.1 |
| Duration of Illness, BD | SMD | Issler et al., 2010 | -0.145 | -0.454, 0.163 | 0.36 | 68.91 | <0.1 |
| Duration of Illness, BD | SMD | Shashidhara et al., 2015 | -0.142 | -0.489, 0.205 | 0.42 | 66.99 | <0.1 |
| Functioning, GAF | SMD | Bener et al., 2016 | -0.385 | -0.645, -0.124 | 0.004 | 0.01 | 0.3 |
| Functioning, GAF | SMD | Centorrino et al., 2006 | -0.456 | -0.637, -0.274 | <0.001 | 0 | 0.82 |
| Functioning, GAF | SMD | Kazhungil et al., 2017 | -0.39 | -0.581, -0.2 | <0.001 | 0.01 | 0.37 |
| Functioning, GAF | SMD | Shashidhara et al., 2015 | -0.427 | -0.624, -0.229 | <0.001 | 0.01 | 0.28 |
| Hospitalization, Number | SMD | Bener et al., 2016 | 0.277 | -0.376, 0.93 | 0.41 | 80.51 | <0.1 |
| Hospitalization, Number | SMD | Kazhungil et al., 2017 | -0.15 | -0.348, 0.048 | 0.14 | 0 | 0.52 |
| Hospitalization, Number | SMD | Shashidhara et al., 2015 | 0.193 | -0.603, 0.988 | 0.64 | 90.19 | <0.1 |
| Manic Episodes, Number | SMD | Jeon et al., 2017 | 0.24 | -0.134, 0.613 | 0.21 | 65.16 | <0.1 |
| Manic Episodes, Number | SMD | de Filippis et al., 2018 | 0.066 | -0.214, 0.346 | 0.64 | 53.38 | <0.1 |
| Manic Episodes, Number | SMD | Kazhungil et al., 2017 | 0.091 | -0.237, 0.418 | 0.59 | 61.72 | <0.1 |
| Manic Episodes, Number | SMD | Issler et al., 2010 | 0.126 | -0.21, 0.463 | 0.46 | 69.72 | <0.1 |
| Manic Episodes, Number | SMD | Ozdemiroglu et al., 2015 | 0.251 | -0.083, 0.585 | 0.14 | 63.59 | <0.1 |
| Manic Episodes, Number | SMD | Shashidhara et al., 2015 | 0.229 | -0.147, 0.606 | 0.23 | 68.72 | <0.1 |
| Mixed Episodes, Number | SMD | Kazhungil et al., 2017 | -0.929 | -3.071, 1.213 | 0.4 | 98.04 | <0.1 |
| Mixed Episodes, Number | SMD | Ozdemiroglu et al., 2015 | -1.162 | -2.847, 0.522 | 0.18 | 96.95 | <0.1 |
| Mixed Episodes, Number | SMD | Shashidhara et al., 2015 | -0.071 | -0.528, 0.386 | 0.76 | 53.48 | 0.14 |
| Symptom Severity, CGI | SMD | Bener et al., 2016 | 0.098 | -0.226, 0.421 | 0.55 | 0 | 0.87 |
| Symptom Severity, CGI | SMD | Centorrino et al., 2006 | -0.386 | -1.281, 0.51 | 0.4 | 93.88 | <0.1 |
| Symptom Severity, CGI | SMD | Shashidhara et al., 2015 | -0.393 | -1.343, 0.558 | 0.42 | 86.8 | <0.1 |
| Symptom Severity, Depression | SMD | Bener et al., 2016 | -0.075 | -0.292, 0.142 | 0.5 | 0 | 0.88 |
| Symptom Severity, Depression | SMD | Braveman et al., 2021 | -0.256 | -0.72, 0.208 | 0.28 | 83.8 | <0.1 |
| Symptom Severity, Depression | SMD | Centorrino et al., 2006 | -0.303 | -0.745, 0.138 | 0.18 | 82.97 | <0.1 |
| Symptom Severity, Depression | SMD | de Filippis et al., 2018 | -0.331 | -0.761, 0.098 | 0.13 | 81.18 | <0.1 |
| Symptom Severity, Depression | SMD | Kazhungil et al., 2017 | -0.296 | -0.757, 0.165 | 0.21 | 81.8 | <0.1 |
| Symptom Severity, Depression | SMD | Shashidhara et al., 2015 | -0.275 | -0.748, 0.198 | 0.26 | 81.33 | <0.1 |
| Symptom Severity, Mania | SMD | Bener et al., 2016 | 0.163 | -0.061, 0.387 | 0.15 | 4.77 | 0.37 |
| Symptom Severity, Mania | SMD | Braveman et al., 2021 | 0.099 | -0.136, 0.333 | 0.41 | 38.44 | 0.18 |
| Symptom Severity, Mania | SMD | Centorrino et al., 2006 | 0.101 | -0.125, 0.326 | 0.38 | 37.02 | 0.2 |
| Symptom Severity, Mania | SMD | de Filippis et al., 2018 | 0.071 | -0.168, 0.311 | 0.56 | 41.17 | 0.15 |
| Symptom Severity, Mania | SMD | Kazhungil et al., 2017 | 0.003 | -0.197, 0.202 | 0.98 | 15.66 | 0.42 |
| Symptom Severity, Mania | SMD | Shashidhara et al., 2015 | 0.007 | -0.209, 0.222 | 0.95 | 19.65 | 0.34 |

## eTable n.7 - Sensitivity analyses, considering only good quality studies

Significant results are in bold.

The results are highlighted in red when the sensitivity analysis does not show significance, while the analysis considering all studies does.

| **Outcome type** | **Studies, n** | **BDOCD patients, n** | **BD patients, n** | **Effect size, type** | **Effect size** | **95% CIs** | **p-value** | **95% PIs** | **I^2^** | **Q test p-value** |
| --- | --- | --- | --- | --- | --- | --- | --- | --- | --- | --- |
| **Adults** |  |  |  |  |  |  |  |  |  |  |
| Affective Episodes, Chronic Course | 2 | 50 | 50 | OR | **9.422** | **2.228, 39.885** | **0.002** | **2.228, 39.885** | 0 | 0.7 |
| Affective Episodes, First episode Depression | 2 | 65 | 401 | OR | 1.042 | 0.47, 2.314 | 0.92 | 0.47, 2.314 | 0 | 0.46 |
| Affective Episodes, First episode Mania | 2 | 65 | 401 | OR | 0.616 | 0.338, 1.124 | 0.11 | 0.338, 1.124 | 0 | 0.76 |
| Affective Episodes, First episode Mixed | 2 | 65 | 401 | OR | 1.931 | 0.799, 4.665 | 0.14 | 0.732, 5.094 | 10.45 | 0.29 |
| Affective Episodes, Psychotic Features | 3 | 80 | 416 | OR | 0.638 | 0.368, 1.103 | 0.11 | 0.368, 1.103 | 0 | 0.61 |
| Affective Episodes, Rapid Cycling | 2 | 67 | 83 | OR | 1.619 | 0.336, 7.807 | 0.55 | 0.183, 14.325 | 45.14 | 0.18 |
| Comorbidity, Agoraphobia | 2 | 122 | 670 | OR | 3.838 | 0.087, 168.511 | 0.49 | 0.008, 1835.367 | 80.89 | <0.1 |
| Comorbidity, Generalized Anxiety Disorder | 4 | 172 | 720 | OR | 1.363 | 0.724, 2.565 | 0.34 | 0.724, 2.565 | 0 | 0.73 |
| Comorbidity, Panic Disorder | 4 | 172 | 720 | OR | 2.026 | 0.978, 4.195 | 0.06 | 0.958, 4.28 | 0.8 | 0.65 |
| Comorbidity, PTSD | 3 | 142 | 354 | OR | 0.665 | 0.296, 1.493 | 0.32 | 0.296, 1.493 | 0 | 0.98 |
| Comorbidity, Social Anxiety Disorder | 4 | 172 | 720 | OR | 1.306 | 0.366, 4.665 | 0.68 | 0.101, 16.844 | 77.3 | <0.1 |
| Comorbidity, Specific Phobia | 3 | 142 | 354 | OR | 1.161 | 0.205, 6.58 | 0.87 | 0.055, 24.656 | 70.26 | <0.1 |
| Comorbidity, Substance Use Disorder | 2 | 65 | 401 | OR | 0.895 | 0.056, 14.253 | 0.94 | 0.013, 62.49 | 64.19 | <0.1 |
| Suicide, Attempt | 5 | 135 | 295 | OR | 1.327 | 0.737, 2.389 | 0.34 | 0.513, 3.435 | 32.51 | 0.23 |
| Age at Onset, BD | 7 | 260 | 976 | SMD | -0.401 | -0.954, 0.152 | 0.16 | -1.884, 1.082 | 91.96 | <0.1 |
| Depressive Episodes, Number | 5 | 135 | 509 | SMD | 0.406 | -0.01, 0.822 | 0.06 | -0.471, 1.283 | 71.4 | <0.1 |
| Duration of Illness, BD | 4 | 163 | 707 | SMD | -0.193 | -0.515, 0.13 | 0.24 | -0.762, 0.377 | 56.1 | <0.1 |
| Functioning, GAF | 3 | 154 | 728 | SMD | **-0.456** | **-0.637, -0.274** | **<0.001** | **-0.637, -0.274** | **0** | 0.82 |
| Hospitalization, Number | 3 | 154 | 728 | SMD | 0.102 | -0.372, 0.575 | 0.67 | -0.778, 0.981 | 82.5 | <0.1 |
| Hypomanic Episodes, Number | 2 | 62 | 414 | SMD | 0.431 | -0.003, 0.864 | 0.05 | -0.195, 1.057 | 53.88 | 0.14 |
| Manic Episodes, Number | 5 | 135 | 509 | SMD | 0.24 | -0.134, 0.613 | 0.21 | -0.522, 1.001 | 65.16 | <0.1 |
| Mixed Episodes, Number | 3 | 94 | 472 | SMD | -0.721 | -2.026, 0.584 | 0.28 | -3.296, 1.853 | 96.45 | <0.1 |
| Symptom Severity, CGI | 2 | 122 | 670 | SMD | -0.386 | -1.281, 0.51 | 0.4 | -1.905, 1.133 | 93.88 | <0.1 |
| Symptom Severity, Depression | 4 | 180 | 750 | SMD | -0.31 | -0.856, 0.236 | 0.27 | -1.46, 0.84 | 87.74 | <0.1 |
| Symptom Severity, Mania | 4 | 180 | 750 | SMD | 0.141 | -0.117, 0.399 | 0.28 | -0.292, 0.574 | 46.28 | 0.14 |

## Publication bias

The funnel plots for each meta-analysis are available at https://osf.io/yc4wd/?view_only=c1f3ef72e6504e7fbc94f91a5336bd97.

## eTable n.8 – Publication bias

| **Age class** | **Outcome type** | **Effect size, type** | **Egger's z** | **p-value** |
| --- | --- | --- | --- | --- |
| Adults | Age at Onset, BD | SMD | -1.964 | 0.049 |
| Adults | Suicide, Attempt | OR | 0.923 | 0.36 |

# Appendix VI

## eTable 9 - PRISMA Checklist

| **1Section and Topic** | **Item #** | **Checklist item** | **Location where item is reported** |
| --- | --- | --- | --- |
| **TITLE** | | |  |
| Title | 1 | Identify the report as a systematic review. | Page 1 |
| **ABSTRACT** | | |  |
| Abstract | 2 | See the PRISMA 2020 for Abstracts checklist. | Page 3 |
| **INTRODUCTION** | | |  |
| Rationale | 3 | Describe the rationale for the review in the context of existing knowledge. | Page 4-5 |
| Objectives | 4 | Provide an explicit statement of the objective(s) or question(s) the review addresses. | Page 4-5 |
| **METHODS** | | |  |
| Eligibility criteria | 5 | Specify the inclusion and exclusion criteria for the review and how studies were grouped for the syntheses. | Page 6 |
| Information sources | 6 | Specify all databases, registers, websites, organisations, reference lists and other sources searched or consulted to identify studies. Specify the date when each source was last searched or consulted. | Page 6 |
| Search strategy | 7 | Present the full search strategies for all databases, registers and websites, including any filters and limits used. | Supp Mat |
| Selection process | 8 | Specify the methods used to decide whether a study met the inclusion criteria of the review, including how many reviewers screened each record and each report retrieved, whether they worked independently, and if applicable, details of automation tools used in the process. | Page 7 |
| Data collection process | 9 | Specify the methods used to collect data from reports, including how many reviewers collected data from each report, whether they worked independently, any processes for obtaining or confirming data from study investigators, and if applicable, details of automation tools used in the process. | Page 6-7 |
| Data items | 10a | List and define all outcomes for which data were sought. Specify whether all results that were compatible with each outcome domain in each study were sought (e.g. for all measures, time points, analyses), and if not, the methods used to decide which results to collect. | Page 6 |
|  | 10b | List and define all other variables for which data were sought (e.g. participant and intervention characteristics, funding sources). Describe any assumptions made about any missing or unclear information. | Page 6-7 |
| Study risk of bias assessment | 11 | Specify the methods used to assess risk of bias in the included studies, including details of the tool(s) used, how many reviewers assessed each study and whether they worked independently, and if applicable, details of automation tools used in the process. | Page 7 |
| Effect measures | 12 | Specify for each outcome the effect measure(s) (e.g. risk ratio, mean difference) used in the synthesis or presentation of results. | Page 7 |
| Synthesis methods | 13a | Describe the processes used to decide which studies were eligible for each synthesis (e.g. tabulating the study intervention characteristics and comparing against the planned groups for each synthesis (item #5)). | Page 6 |
|  | 13b | Describe any methods required to prepare the data for presentation or synthesis, such as handling of missing summary statistics, or data conversions. | Page 7-8 |
|  | 13c | Describe any methods used to tabulate or visually display results of individual studies and syntheses. | Page 7 |
|  | 13d | Describe any methods used to synthesize results and provide a rationale for the choice(s). If meta-analysis was performed, describe the model(s), method(s) to identify the presence and extent of statistical heterogeneity, and software package(s) used. | Page 7-8 |
|  | 13e | Describe any methods used to explore possible causes of heterogeneity among study results (e.g. subgroup analysis, meta-regression). | Page 7-8 |
|  | 13f | Describe any sensitivity analyses conducted to assess robustness of the synthesized results. | Page 7-8 |
| Reporting bias assessment | 14 | Describe any methods used to assess risk of bias due to missing results in a synthesis (arising from reporting biases). | Page 7-8 |
| Certainty assessment | 15 | Describe any methods used to assess certainty (or confidence) in the body of evidence for an outcome. | NA |
| **RESULTS** | | |  |
| Study selection | 16a | Describe the results of the search and selection process, from the number of records identified in the search to the number of studies included in the review, ideally using a flow diagram. | Page 9, Figure 1 |
|  | 16b | Cite studies that might appear to meet the inclusion criteria, but which were excluded, and explain why they were excluded. | Supp Mat |
| Study characteristics | 17 | Cite each included study and present its characteristics. | Page 9-11, Table 1 |
| Risk of bias in studies | 18 | Present assessments of risk of bias for each included study. | Page 9, Table 1, Supp Mat |
| Results of individual studies | 19 | For all outcomes, present, for each study: (a) summary statistics for each group (where appropriate) and (b) an effect estimate and its precision (e.g. confidence/credible interval), ideally using structured tables or plots. | Page 9-11, Figure 2, Table 2, Supp Mat |
| Results of syntheses | 20a | For each synthesis, briefly summarise the characteristics and risk of bias among contributing studies. | Page 9-11, Figure 2, Table 2, Supp Mat |
|  | 20b | Present results of all statistical syntheses conducted. If meta-analysis was done, present for each the summary estimate and its precision (e.g. confidence/credible interval) and measures of statistical heterogeneity. If comparing groups, describe the direction of the effect. | Page 9-11, Figure 2, Table 2, Supp Mat |
|  | 20c | Present results of all investigations of possible causes of heterogeneity among study results. | Page 9-11, Figure 2, Table 2, Supp Mat |
|  | 20d | Present results of all sensitivity analyses conducted to assess the robustness of the synthesized results. | Page 9-11, Figure 2, Table 2, Supp Mat |
| Reporting biases | 21 | Present assessments of risk of bias due to missing results (arising from reporting biases) for each synthesis assessed. | Page 9-11, Figure 2, Table 2, Supp Mat |
| Certainty of evidence | 22 | Present assessments of certainty (or confidence) in the body of evidence for each outcome assessed. | NA |
| **DISCUSSION** | | |  |
| Discussion | 23a | Provide a general interpretation of the results in the context of other evidence. | Page 12-16 |
|  | 23b | Discuss any limitations of the evidence included in the review. | Page 15 |
|  | 23c | Discuss any limitations of the review processes used. | Page 15 |
|  | 23d | Discuss implications of the results for practice, policy, and future research. | Page 14-16 |
| **OTHER INFORMATION** | | |  |
| Registration and protocol | 24a | Provide registration information for the review, including register name and registration number, or state that the review was not registered. | Page 6 |
|  | 24b | Indicate where the review protocol can be accessed, or state that a protocol was not prepared. | Page 6 |
|  | 24c | Describe and explain any amendments to information provided at registration or in the protocol. | Page 6, Supp Mat |
| Support | 25 | Describe sources of financial or non-financial support for the review, and the role of the funders or sponsors in the review. | Page 17 |
| Competing interests | 26 | Declare any competing interests of review authors. | Page 17 |
| Availability of data, code and other materials | 27 | Report which of the following are publicly available and where they can be found: template data collection forms; data extracted from included studies; data used for all analyses; analytic code; any other materials used in the review. | Page 17 |
